# False discovery rate estimation using candidate peptides for each spectrum

**DOI:** 10.1186/s12859-022-05002-4

**Published:** 2022-11-01

**Authors:** Sangjeong Lee, Heejin Park, Hyunwoo Kim

**Affiliations:** 1 Department of Computer Science, Hanyang University, Seoul, 06978 Republic of Korea; 2 Biomedical Informatics Team, Korea Institute of Science and Technology Information, Daejeon, 34141 Republic of Korea

**Keywords:** Target-decoy strategy, False discovery rate, Proteomics, Tandem mass spectrometry

## Abstract

**Background:**

False discovery rate (FDR) estimation is very important in proteomics. The target-decoy strategy (TDS), which is often used for FDR estimation, estimates the FDR under the assumption that when spectra are identified incorrectly, the probabilities of the spectra matching the target or decoy peptides are identical. However, no spectra matching target or decoy peptide probabilities are identical. We propose cTDS (target-decoy strategy with candidate peptides) for accurate estimation of the FDR using the probability that the spectrum is identified incorrectly as a target or decoy peptide.

**Results:**

Most spectrum cases result in a probability of having the spectrum identified incorrectly as a target or decoy peptide of close to 0.5, but only about 1.14–4.85% of the total spectra have an exact probability of 0.5. We used an entrapment sequence method to demonstrate the accuracy of cTDS. For fixed FDR thresholds (1–10%), the false match rate (FMR) in cTDS is closer than the FMR in TDS. We compared the number of peptide-spectrum matches (PSMs) obtained with TDS and cTDS at a 1% FDR threshold with the HEK293 dataset. In the first and third replications, the number of PSMs obtained with cTDS for the reverse, pseudo-reverse, shuffle, and de Bruijn databases exceeded those obtained with TDS (about 0.001–0.132%), with the pseudo-shuffle database containing less compared to TDS (about 0.05–0.126%). In the second replication, the number of PSMs obtained with cTDS for all databases exceeds that obtained with TDS (about 0.013–0.274%).

**Conclusions:**

When spectra are actually identified incorrectly, most probabilities of the spectra matching a target or decoy peptide are not identical. Therefore, we propose cTDS, which estimates the FDR more accurately using the probability of the spectrum being identified incorrectly as a target or decoy peptide.

**Supplementary Information:**

The online version contains supplementary material available at 10.1186/s12859-022-05002-4.

## Background

Liquid chromatography and tandem mass spectrometry (LC–MS/MS) in shotgun proteomics are methods that can be used to analyze protein and peptides [1]. Spectra generated by LC–MS/MS are identified as peptides using various methods, such as a database search [2–4], de novo sequencing [5–7], or with a spectral library [8–11]. However, a large set of peptide-spectrum matches (PSMs) identified by various search tools causes a quality assessment problem when making multiple comparisons [12, 13]. For this reason, it is very important to estimate the false discovery rate (FDR) properly in proteomics.

The target-decoy strategy (henceforth TDS) is a simple approach that is frequently used to estimate the FDR in proteomics [14, 15]. This strategy effectively estimates the FDR with the generated target and decoy PSMs by searching for the spectra in a target-decoy database. To support an accurate FDR estimation, various methods have been proposed, such as decoy database creation methods that normally construct a decoy database by reversing or shuffling the target protein database [16–19], FDR estimation methods that rely on the creation method [20, 21], and a post-processing method that re-ranks the PSM list obtained by a database search algorithm and then sets a threshold automatically in the re-ranked list using a specific method [22].

One of the assumptions associated with TDS is that “target and decoy false positives are equally likely.” This assumption has been demonstrated to hold under certain conditions in the following ways [15]: (1) the ratio of the top-ranked target and decoy PSMs matched after shifting the precursor mass of the input MS/MS spectra (these spectra are always incorrect PSMs), (2) the ratio of low-ranked target and decoy PSMs on the PSM list (using rank 2–5 PSMs), and (3) the ratio of unique peptides in target and decoy databases. TDS is a means of estimating the FDR using target and decoy PSMs. Additionally, when using stochastic decoy databases such as shuffle and random decoy databases or when the sizes of the target and decoy databases are not the same, the probability that an incorrect PSM matches a target or decoy peptide may not be the same. To solve this problem, the FDR is estimated with a correction factor [14, 15, 18].

TDS estimates the FDR upon the assumption that when all spectra are identified incorrectly, the probabilities of the spectra matching the target or decoy peptides are identical. However, when the spectrum is identified incorrectly, the probabilities of the spectra matching the target or decoy peptides are not the same. If the probabilities of the spectra matching the target or decoy peptides are not equal, the estimate of the FDR can be inaccurate. The probability of a single spectrum being identified incorrectly as a target or decoy peptide can be calculated using the number of target and decoy candidate peptides (Eq. 5 in the Methods section). Therefore, we propose cTDS (target-decoy strategy with candidate peptides), which is a new method to estimate the FDR using the probabilities of a single spectrum identified incorrectly as a target or decoy peptide. We calculate this ratio using the number of target and decoy candidate peptides corresponding to a specific spectrum and conduct an entrapment experiment [23, 24] to demonstrate the accuracy of cTDS. Finally, we compared FDR results estimated with both the TDS and cTDS methods.

## Results

For convenience, we denote the concatenated database (target + decoy databases) with R meaning reverse, PR meaning pseudo-reverse, S meaning shuffle, PS meaning pseudo-shuffle, and DE referring to the de Bruijn decoy database. (S and PS are different every time we generate decoy databases when using the shuffle method. Therefore, S and PS S1-4 and PS1-4 show variations of S and PS; the replicates of each dataset are denoted as R1, R2, and R3.)

### Ratio of target and decoy candidate peptides corresponding to a specific spectrum

**Fig. 1:**
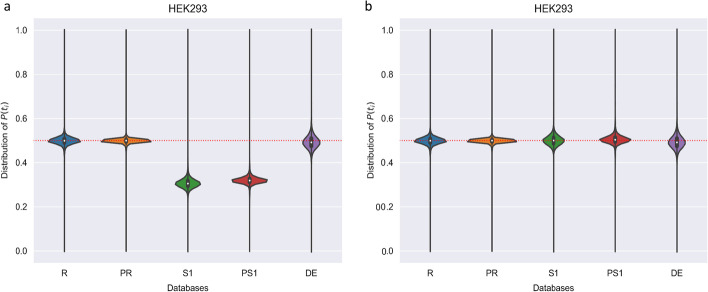
Comparison of the distributions of $\mathrm{P}({t}_{i})$ in various databases and in the HEK293 dataset. The x-axis represents the ratio of PSMs with each $\mathrm{P}({t}_{i})$. The y-axis represents different databases: **a** without a correction factor, and **b** with a correction factor

First, we examined the ratio of the number of target and decoy candidate peptides for each spectrum to discern whether the probabilities of the spectra matching the target or decoy peptides are not the same when the spectra are identified incorrectly (Fig. 1 and Additional file 1: Fig. S1). Figure 1 shows the distribution of $\mathrm{P}({t}_{i})$ for each spectrum in the HEK293 dataset. $\mathrm{P}({t}_{i})$ represents the ratio of the target candidate peptides among the target and decoy candidate peptides corresponding to a specific spectrum (See Eq. 5 in the Methods section). In Fig. 1a, R, PR, and DE are distributed close to 0.5, while S and PS are distributed at different positions because for R, PR, and DE, the sizes of the target and decoy database are nearly identical, whereas for S and PS, this is not the case. Figure 1b shows when a correction factor is applied to correct the distributions of S and PS. As shown in Fig. 1b, most of the spectrum has a $\mathrm{P}({t}_{i})$ value close to 0.5, while only about 1.14–4.85% of the total spectrum has a $\mathrm{P}({t}_{i})$ value of exactly 0.5 (R, PR, S, PS, and DE). In conclusion, when the spectra in the HEK293 dataset are identified incorrectly, the probabilities of the spectra matching the target or decoy peptides are mostly not equal. This also applies to other cell line datasets (Additional file 1: Fig. S2).

### Accuracy comparisons using an entrapment sequence

We used an entrapment experiment to demonstrate the accuracy of cTDS. The entrapment sequence method uses an entrapment database that is added to a reference database to create the target database. After estimating the FDR, a PSM matched to the reference database is classified as a true positive and a PSM matched to the entrapment database is classified as a false positive. Then, the false match ratio (FMR) is calculated using Eq. 4 in the Methods section.

**Fig. 2:**
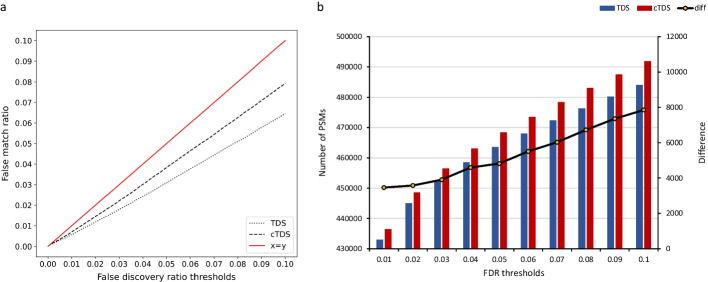
Comparison of the FMR and number of PSMs at fixed FDR thresholds. **a** The dashed line represents the FMR in cTDS. The dotted line represents the FMR in TDS. The x-axis and y-axis represent the false discovery ratio thresholds and the false match ratio, respectively. **b** The x-axis represents the FDR thresholds. The y-axis represents the number of PSMs

To compare TDS and cTDS, we generated a target database as a human synthetic peptide database (reference database) combined with the *P. furiosus* database (entrapment database). Subsequently, a reverse decoy database was concatenated into the created target database. Synthetic peptide datasets, which are actual ground truth datasets for which measurements are more accurate, were then searched by applying the same parameters described in the Methods section. Figure 2a compares the FMR outcomes for TDS and cTDS at fixed FDR thresholds. As shown in Fig. 2a, for fixed FDR thresholds (1–10%), the FMR in cTDS is closer than FMR in TDS. This result indicates that TDS is more conservative than cTDS. Figure 2b shows the number of PSMs obtained with TDS and cTDS at fixed FDR thresholds. The blue bar and the red bar represent the number of PSMs obtained by TDS and cTDS, respectively, and the black line represents the difference in the number of PSMs between TDS and cTDS. As shown in Fig. 2b, the number of PSMs obtained with cTDS exceeds those by TDS (by approximately 0.799–1.621%) at all FDR thresholds.

### Performance capabilities of TDS and cTDS

**Fig. 3:**
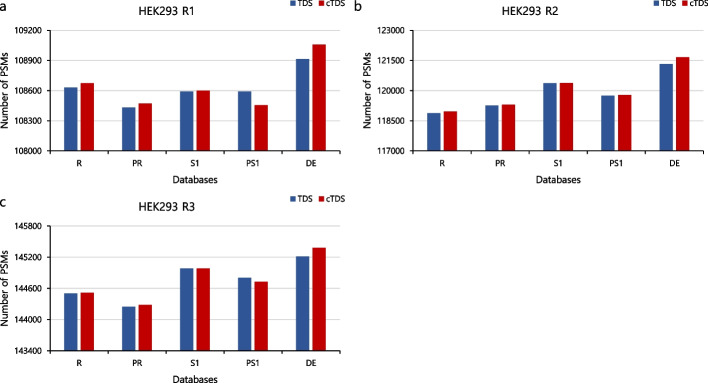
Comparison of the number of PSMs of various databases. The blue bar shows the number of PSMs obtained with TDS at the 1% FDR threshold. The red bar shows the number of PSMs obtained with cTDS at the 1% FDR threshold: **a** HEK293 first replicate, **b** HEK293 second replicate, and **c** HEK293 third replicate

We compared the newly proposed cTDS to TDS. Figure 3 shows the number of PSMs obtained with TDS and cTDS at a 1% FDR threshold using the HEK293 dataset and various databases. The blue bar and the red bar represent the number of PSMs obtained with TDS and cTDS, respectively. With TDS, the stochastic methods S and PS utilized a correction factor, which is the ratio of the rank 5 target and decoy PSMs. As shown in Fig. 3 (Additional file 1: Fig. S3), in the first and third replications, the number of PSMs obtained with cTDS for R, PR, S, and DE exceed those by TDS (about 0.001–0.132%), with the PS containing fewer PSMs compared to TDS (about 0.05–0.126%). In the second replication, the number of PSMs obtained with cTDS for all databases exceeds those obtained with TDS (about 0.013–0.274%). (Additional file 1: Figs. S4 and S5 show the variations of S and PS for the other ten datasets used here.)

**Fig. 4:**
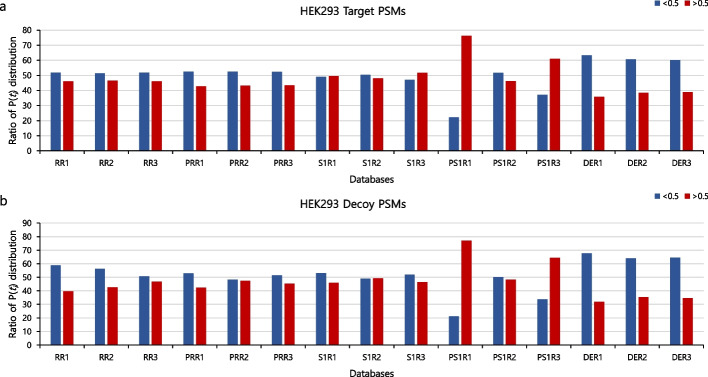
Comparison of the ratio of $\mathrm{P}({t}_{i})$ distributions of target and decoy hits in various databases and in the HEK293 dataset. The blue bar shows the ratio of spectra for which $\mathrm{P}({t}_{i})$ < 0.5 among all spectra at the 1% FDR threshold. The red bar shows the ratio of spectra for which $\mathrm{P}({t}_{i})$ > 0.5 among all spectra at the 1% FDR threshold: **a** HEK293 target hits, and **b** HEK293 decoy hits

When comparing TDS and cTDS, the identified spectra increase or decrease because the distribution of $\mathrm{P}({t}_{i})$ for each spectrum differs. Figure 4 shows the ratio of the distribution of $\mathrm{P}({t}_{i})$ of the identified spectra obtained for R, PR, S, PS, and DE and the HEK293 dataset at the 1% FDR threshold. The blue bar represents the case of $\mathrm{P}({t}_{i})$ < 0.5 and the red bar denotes $\mathrm{P}({t}_{i})$ > 0.5. In other words, the blue bar indicates that decoy candidate peptides outnumber the target candidate peptides among all candidate peptides for each spectrum, and the red bar indicates the opposite. Figure 4a presents the distribution of $\mathrm{P}({t}_{i})$ of the spectra identified as target peptides, and Fig. 4b is the distribution of $\mathrm{P}({t}_{i})$ of the spectra identified as decoy peptides.

In Fig. 4a, b, when the blue bar is higher than the red bar, it indicates that the identified spectrum has more decoy candidate peptides than target candidate peptides, meaning that the incorrectly identified spectrum is highly likely to be identified as a decoy peptide. Consequently, the number of identified spectra with cTDS at the 1% FDR threshold increases, as shown in Fig. 3 (R, PR, and DE in the first replicate; R, PR, PS, and DE in the second replicate; and R, PR, and DE in the third replicate). Conversely, in Fig. 4a, b, when the red bar is higher than the blue bar, the identified spectrum has more target candidate peptides than decoy candidate peptides. This indicates that an incorrectly identified spectrum is highly likely to be identified as a target peptide. Consequently, the number of identified spectra with cTDS at the 1% FDR threshold decreases, as shown in Fig. 3 (PS at the first and third replicates).

However, for S shown in Fig. 4a, b, neither the blue bar nor the red bar is high for the three replicates. For example, in the first replicate, the red bar is taller than the blue bar in Fig. 4a, and the blue bar is taller than the red bar in Fig. 4b. In this case, we do not know whether TDS estimated the FDR correctly. The proposed cTDS estimates the FDR by thoroughly considering all of these cases. When the FDR was estimated with cTDS, with regard to S, the number of identified spectra increased for all three replications. (In addition, see Additional file 1: Figs. S6 and S7 for variations of S and PS with the other ten datasets.)

## Discussion

Various search methods and FDR estimation methods have been proposed for proteomics. Here, we propose cTDS, which uses the probabilities of spectra being identified incorrectly as target or decoy peptides, to estimate a more accurate FDR. Compared to TDS, cTDS estimates the FDR more accurately. With TDS, when the sizes of the target and decoy databases are different, such as in protein-level shuffle or pseudo-shuffle decoy databases, the FDR should be estimated using a correction factor. However, cTDS can estimate the FDR without a correction factor regardless of which decoy database is used. When generating a peptide-level decoy database, cTDS is identical to the existing TDS. However, when generating a protein-level decoy database, cTDS can estimate the FDR more accurately than TDS.

For cTDS, the FDR can be estimated when the number of target and decoy candidate peptides is known. The FDR can easily be estimated according to the output of the number of target and decoy candidate peptides using existing database search tools. However, most existing database search tools do not present the numbers of target and decoy candidate peptides as the output. Therefore, in order to estimate the FDR more accurately, we hope the method used is supported by the output of the numbers of target and decoy candidate peptides when developing database search tools. We modified Comet to print the number of candidate peptides. The modified code is available at https://github.com/othertics/CometCandidateCount.

## Conclusion

The most commonly used method to estimate the FDR is the TDS method. TDS estimates the FDR under the assumption that when all spectra are identified incorrectly, the probabilities of the spectra matching the target or decoy peptides are identical. However, when spectra are actually identified incorrectly, most of the probabilities of the spectra matching the target or decoy peptides are not the same. This problem complicates accurate FDR estimation. Therefore, we proposed cTDS, which estimates the FDR more accurately using the probabilities of spectra identified incorrectly as target or decoy peptides. It is demonstrated that cTDS shows no substantial differences in terms of the number of identified spectra compared to TDS, but it can be said to be a more accurate FDR estimation method.

## Methods

### Datasets and parameters

An MS/MS dataset compiled from eleven cell lines (A549, GAMG, HEK293, HeLa, HepG2, Jurkat, K562, LnCap, MCF7, RKO, and U2OS) and the synthetic peptides were obtained with an LTQ-Orbitrap Velos mass spectrometer and an Orbitrap Fusion Lumos mass spectrometer (Thermo Fisher Scientific, Bremen, Germany) [25, 26] (See Additional file 1: Tables S1 and S2 for detailed information). We used the synthetic peptide datasets to demonstrate the accuracy of cTDS because it is a set of spectra generated from actual synthetic peptides. Therefore, if a spectrum matches a peptide that does not exist in the synthetic peptide database, it is considered to be incorrect. Hence, we used a synthetic peptide dataset, an actual ground truth dataset to distinguish true and false positives. We also searched with Comet (2019.01 rev. 1 version) with the following high-resolution parameters: precursor tolerance = 20 ppm, fragment tolerance = 0.02 Da, number of tryptic termini = 2, maximum missed cleavages = 2, fixed modification = carbamidomethyl on cysteine, and variable modification = oxidation on methionine.

### Databases

We used the human SwissProt database (42,351 target proteins, 2,618,539 target peptides), a synthetic peptide database (12,622 target peptides), and the Pyrococcus furiosus (*P. furiosus*) UniProt database (992 target proteins, 77,661 target peptides) to compare the results estimated with TDS and cTDS at a fixed FDR threshold. The synthetic peptide database is a list of peptides used for synthesis in the ProteomeTools project.

### Decoy database generation

#### Reverse method

This method generates a decoy database by reversing the proteins of a given target database. For example, for the target protein “GCNKYQWR,” the decoy protein “RWQYKNCG” is generated by reversing the target protein as it is.

#### Pseudo-reverse method

This method generates in a manner identical to that of the reverse method, but it reverses only the peptides between K and R. For example, for the target protein “GCNKYQWR,” the decoy protein “NCGKWQYR” is generated by reversing the peptides between K and R of the target protein.

#### Shuffle method

This method generates a decoy database by shuffling the protein of a given target database. For example, for the target protein “GCNKYQWR,” the decoy protein “NKYQCWGR” is generated by shuffling the target protein as it is.

#### Pseudo-shuffle method

The generation process by this method is identical to that of the shuffle method, but it shuffles only the peptides between K and R. For example, for the target protein “GCNKYQWR,” the decoy protein “CNGKYWQR” is generated by shuffling the peptides between K and R of the target protein.

#### De Bruijn method

This method generates a decoy database with a de Bruijn graph for a given target database. For example, for the target protein “GCNKYQWR,” the target protein is transformed into a k-mer form and a graph is implemented. The decoy protein “NGCKWYQR” is then generated by altering the edges representing the amino acid according to the amino acid probabilities of the target database.

### Target-decoy strategy

TDS FDR estimates are done using the method expressed by Eq. 1, as shown below.

1 $${\mathrm{FDR}}_{TDS}= \frac{D+1}{T}$$

Here, $T$ is the number of target PSMs and $D$ is the number of decoy PSMs. Additionally, when the sizes of the target and decoy databases are different, such as in protein-level shuffle or pseudo shuffle decoy databases, the FDR can be estimated using the method expressed by Eq. 2 below.

2 $${FDR}_{TDS}= \frac{D+1}{T} \times factor$$

In this equation, $factor$ is a value used to correct the FDR estimation when the sizes of the target and decoy databases differ. In this paper, $factor$ is the ratio of the rank 5 target and decoy PSMs. It is calculated using the equation below.

3 $$factor= \frac{\#Target}{\#Decoy}$$

Here, $\#Target$ is the number of target PSMs of rank 5, and $\#Decoy$ is the number of decoy PSMs of rank 5.

### False match rate

FMR is a method for approximatively estimating false positives at a specific FDR threshold. FMR is calculated by Eq. 4 below.

4 $$FMR= \frac{{\#Target}_{entrap}}{{\#Target}_{refer}}$$

In this equation, ${\#Target}_{entrap}$ is the number of target PSMs that matched the entrapment database, and ${\#Target}_{refer}$ is the number of target PSMs that matched the reference database.

### False discovery rate estimation using candidate peptides for each spectrum

When a spectrum is identified incorrectly, we estimate the FDR using the probability that it will be identified as a target peptide and the probability that it will be identified as a decoy peptide. When $S (S=\left\{{S}_{0}, {S}_{1}, \dots ,{S}_{n}\right\})$ is a set of *n* spectra, $P\left({t}_{i}\right)$ denotes the probability of spectrum ${S}_{i}$ being identified as a target peptide, and $P\left({d}_{i}\right)$ represents the probability of ${S}_{i}$ being identified as a decoy peptide. The method used to calculate $P({t}_{i})$ and $P({d}_{i})$ is as follows.

5 $${P(t}_{i})= \frac{{target}_{i}}{{target}_{i}+ {decoy}_{i}} , \quad {P(d}_{i})= \frac{{decoy}_{i}}{{target}_{i}+ {decoy}_{i}}$$

In these equations, ${target}_{i}$ is the number of target candidate peptides of ${S}_{i}$ and ${decoy}_{i}$ is the number of decoy candidate peptides of ${S}_{i}$. When a single spectrum is identified incorrectly, *X* is a geometric random variable representing the number of experiments conducted before the matching of a target peptide; when a single spectrum is identified incorrectly, only two types of target or decoy peptides are matched, and the probability of the spectrum being matched as a target peptide is always identical to ${t}_{i}$ and is independent every time. When a single spectrum is identified incorrectly, *Y*, like $X$, is a geometric random variable that indicates the number of experiments conducted before the matching of a decoy peptide. We estimate the FDR using the expected values of $E({X}_{i})$ and $E({Y}_{i})$ of the geometric random variable with the equation below.

6 $${\mathrm{FDR}}_{cTDS} = \frac{\sum_{{S}_{i} \in D}E({Y}_{i})}{\sum_{{S}_{i} \in T}E({X}_{i}) } = \frac{\sum_{{S}_{i} \in D}\frac{1}{P({d}_{i})}}{\sum_{{S}_{i} \in T}\frac{1}{P({t}_{i})}}$$

Here, ${S}_{i}\in T$ indicates that ${S}_{i}$ is identified as a target peptide and ${S}_{i}\in D$ signifies that ${S}_{i}$ is identified as a decoy peptide. ${X}_{i}$ and ${Y}_{i}$ are geometric random variables of ${S}_{i}$.

## Supplementary Information

**Additional file 1:** Supplementary tables and figures.

## Data Availability

The eleven human cell lines (A549, GAMG, HEK293, HeLa, HepG2, Jurkat, K562, LnCap, MCF7, RKO and U2OS, each 3 replicates) dataset is publicly available from https://www.ebi.ac.uk/pride-/archive/ using PXD002395. The synthetic peptides dataset is publicly available from https://www.ebi.ac.uk/pride/archive/projects/ using PXD004732. The modified code of Comet is available at https://github.com/othertics/CometCandidateCount.

## Abbreviations

LC–MS/MS: Liquid chromatography and tandem mass spectrometry
FDR: False discovery rate
TDS: Target-decoy strategy
PSMs: Peptide-spectrum matches
FMR: False match rate
R: Target-reverse decoy database
PR: Target-pseudo reverse decoy database
S: Target-shuffle decoy database
PS: Target-pseudo shuffle decoy database
DE: Target-de Bruijn decoy database

## References

[1] Steen H, Mann M (2004). The ABC's (and XYZ's) of peptide sequencing. Nat Rev Mol Cell Biol.

[2] Eng JK, McCormack AL, Yates JR (1994). An approach to correlate tandem mass spectral data of peptides with amino acid sequences in a protein database. J Am Soc Mass Spectrom.

[3] Perkins DN, Pappin DJ, Creasy DM, Cottrell JS (1999). Probability-based protein identification by searching sequence databases using mass spectrometry data. Electrophoresis.

[4] Kim S, Gupta N, Pevzner PA (2008). Spectral probabilities and generating functions of tandem mass spectra: a strike against decoy databases. J Proteome Res.

[5] Dancik V, Addona TA, Clauser KR, Vath JE, Pevzner PA (1999). De novo peptide sequencing via tandem mass spectrometry. J Comput Biol.

[6] Ma B, Zhang K, Hendrie C, Liang C, Li M, Doherty-Kirby A, Lajoie G (2003). PEAKS: powerful software for peptide de novo sequencing by tandem mass spectrometry. Rapid Commun Mass Spectrom.

[7] Frank A, Pevzner P (2005). PepNovo: de novo peptide sequencing via probabilistic network modeling. Anal Chem.

[8] Yates JR, Morgan SF, Gatlin CL, Griffin PR, Eng JK (1998). Method to compare collision-induced dissociation spectra of peptides: potential for library searching and subtractive analysis. Anal Chem.

[9] Craig R, Cortens JC, Fenyo D, Beavis RC (2006). Using annotated peptide mass spectrum libraries for protein identification. J Proteome Res.

[10] Frewen BE, Merrihew GE, Wu CC, Noble WS, MacCoss MJ (2006). Analysis of peptide MS/MS spectra from large-scale proteomics experiments using spectrum libraries. Anal Chem.

[11] Nesvizhskii AI (2010). A survey of computational methods and error rate estimation procedures for peptide and protein identification in shotgun proteomics. J Proteomics.

[12] Granholm V, Kall L (2011). Quality assessments of peptide-spectrum matches in shotgun proteomics. Proteomics.

[13] Levitsky LI, Ivanov MV, Lobas AA, Gorshkov MV (2017). Unbiased false discovery rate estimation for shotgun proteomics based on the target-decoy approach. J Proteome Res.

[14] Elias JE, Gygi SP (2007). Target-decoy search strategy for increased confidence in large-scale protein identifications by mass spectrometry. Nat Methods.

[15] Elias JE, Gygi SP (2010). Target-decoy search strategy for mass spectrometry-based proteomics. Methods Mol Biol.

[16] Wang G, Wu WW, Zhang Z, Masilamani S, Shen RF (2009). Decoy methods for assessing false positives and false discovery rates in shotgun proteomics. Anal Chem.

[17] Jeong K, Kim S, Bandeira N (2012). False discovery rates in spectral identification. BMC Bioinform.

[18] Kim H, Lee S, Park H (2019). Target-small decoy search strategy for false discovery rate estimation. BMC Bioinform.

[19] Moosa JM, Guan S, Moran MF, Ma B (2020). Repeat-preserving decoy database for false discovery rate estimation in peptide identification. J Proteome Res.

[20] Keich U, Tamura K, Noble WS (2019). Averaging strategy to reduce variability in target-decoy estimates of false discovery rate. J Proteome Res.

[21] Gupta N, Bandeira N, Keich U, Pevzner PA (2011). Target-decoy approach and false discovery rate: when things may go wrong. J Am Soc Mass Spectr.

[22] Kall L, Canterbury JD, Weston J, Noble WS, MacCoss MJ (2007). Semi-supervised learning for peptide identification from shotgun proteomics datasets. Nat Methods.

[23] Granholm V, Navarro JF, Noble WS, Kall L (2013). Determining the calibration of confidence estimation procedures for unique peptides in shotgun proteomics. J Proteomics.

[24] Feng XD, Li LW, Zhang JH, Zhu YP, Chang C, Shu KX, Ma J (2017). Using the entrapment sequence method as a standard to evaluate key steps of proteomics data analysis process. BMC Genom.

[25] Geiger T, Wehner A, Schaab C, Cox J, Mann M. Comparative proteomic analysis of eleven common cell lines reveals ubiquitous but varying expression of most proteins. Mol Cell Proteomics 2012, 11(3):M111.014050. 10.1074/mcp.M111.014050 PMC3316730 22278370

[26] Zolg DP, Wilhelm M (2017). Building ProteomeTools based on a complete synthetic human proteome. Nat Methods.

