# Supplementary material for: False discovery rate estimation using candidate peptides for each spectrum
Source: BMC Bioinformatics. 2022 Nov 1;23:454. doi: 10.1186/s12859-022-05002-4 (PMC9623924; doi:10.1186/s12859-022-05002-4)
Supplement: Supplementary file 1 — Additional file 1: Supplementary tables and figures. [file 12859_2022_5002_MOESM1_ESM.docx]

**False discovery rate estimation using candidate peptides for each spectrum**

**Supporting Information**

Sangjeong Lee^1^, Heejin Park^1^, Hyunwoo Kim^2,*^

^1^ Department of Computer Science, Hanyang University, Seoul 06978, Republic of Korea

^2^ Biomedical Informatics Team, Korea Institute of Science and Technology Information, Daejeon 34141, Republic of Korea

Email:

Sangjeong Lee: othertics@hanyang.ac.kr

Heejin Park: hjpark@hanyang.ac.kr

Hyunwoo Kim: pardess@kisti.re.kr

*Corresponding author.

Hyunwoo Kim, Email: [pardess@kisti.re.kr](mailto:pardess@kisti.re.kr)

**Supplementary Table 1. Number of MS/MS Spectra for Each Dataset**

|  | 1^st^ replicate | 2^nd^ replicate | 3^rd^ replicate | Number of total MS/MS spectra |
| --- | --- | --- | --- | --- |
| A549 | 136,309 | 238,468 | 140,135 | 514,912 |
| GAMG | 152,777 | 252,249 | 220,325 | 625,351 |
| HEK293 | 148,800 | 254,686 | 220,622 | 624,108 |
| HeLa | 159,455 | 208,438 | 175,870 | 543,763 |
| HepG2 | 149,974 | 202,529 | 159,455 | 511,958 |
| Jurkat | 160,225 | 200,004 | 210,840 | 571,069 |
| K562 | 167,429 | 159,346 | 127,837 | 454,612 |
| LnCap | 161,334 | 254,323 | 224,270 | 639,927 |
| MCF7 | 174,709 | 240,504 | 225,317 | 640,530 |
| RKO | 164,317 | 212,051 | 125,711 | 502,079 |
| U2OS | 165,271 | 227,369 | 248,574 | 641,214 |

**Supplementary Table 2. Number of MS/MS Spectra for Synthetic Dataset**

|  | Number of  MS/MS spectra |  | Number of  MS/MS spectra |
| --- | --- | --- | --- |
| 01625b_GA1-TUM_first_pool_1_01_01-3xHCD-1h-R1 | 48,417 | 01625b_GB1-TUM_first_pool_2_01_01-3xHCD-1h-R1 | 45,687 |
| 01625b_GA2-TUM_first_pool_9_01_01-3xHCD-1h-R1 | 50,535 | 01625b_GB2-TUM_first_pool_10_01_01-3xHCD-1h-R1 | 48,192 |
| 01625b_GA3-TUM_first_pool_17_01_01-3xHCD-1h-R1 | 52,257 | 01625b_GB3-TUM_first_pool_18_01_01-3xHCD-1h-R1 | 52,227 |
| 01625b_GA4-TUM_first_pool_25_01_01-3xHCD-1h-R1 | 53,748 | 01625b_GB4-TUM_first_pool_26_01_01-3xHCD-1h-R1 | 54,561 |
| 01625b_GA5-TUM_first_pool_33_01_01-3xHCD-1h-R1 | 50,739 | 01625b_GB5-TUM_first_pool_34_01_01-3xHCD-1h-R1 | 50,523 |
| 01625b_GA6-TUM_first_pool_41_01_01-3xHCD-1h-R1 | 54,567 | 01625b_GB6-TUM_first_pool_42_01_01-3xHCD-1h-R1 | 54,393 |
| 01625b_GA7-TUM_first_pool_120_01_01-3xHCD-1h-R1 | 46,071 | 01625b_GB7-TUM_first_pool_121_01_01-3xHCD-1h-R1 | 42,753 |
| Number of total spectra | 704,670 | | |

**
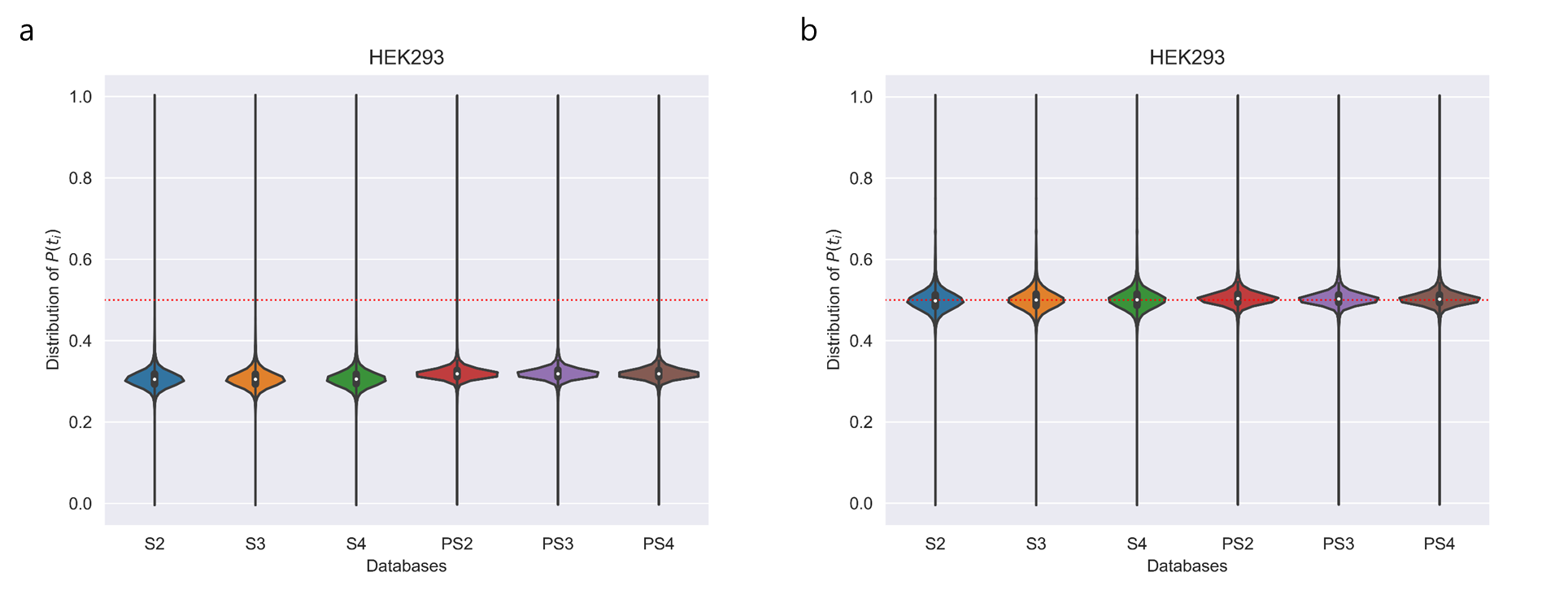
**

**Supplementary Figure 1.** Comparison of the distributions of $P(t_{i})$ for the stochastic databases and for the HEK293 dataset. The x-axis represents the ratio of PSM with each $P(t_{i})$. The y-axis represents different databases: (a) without a correction factor, and (b) with a correction factor.

**
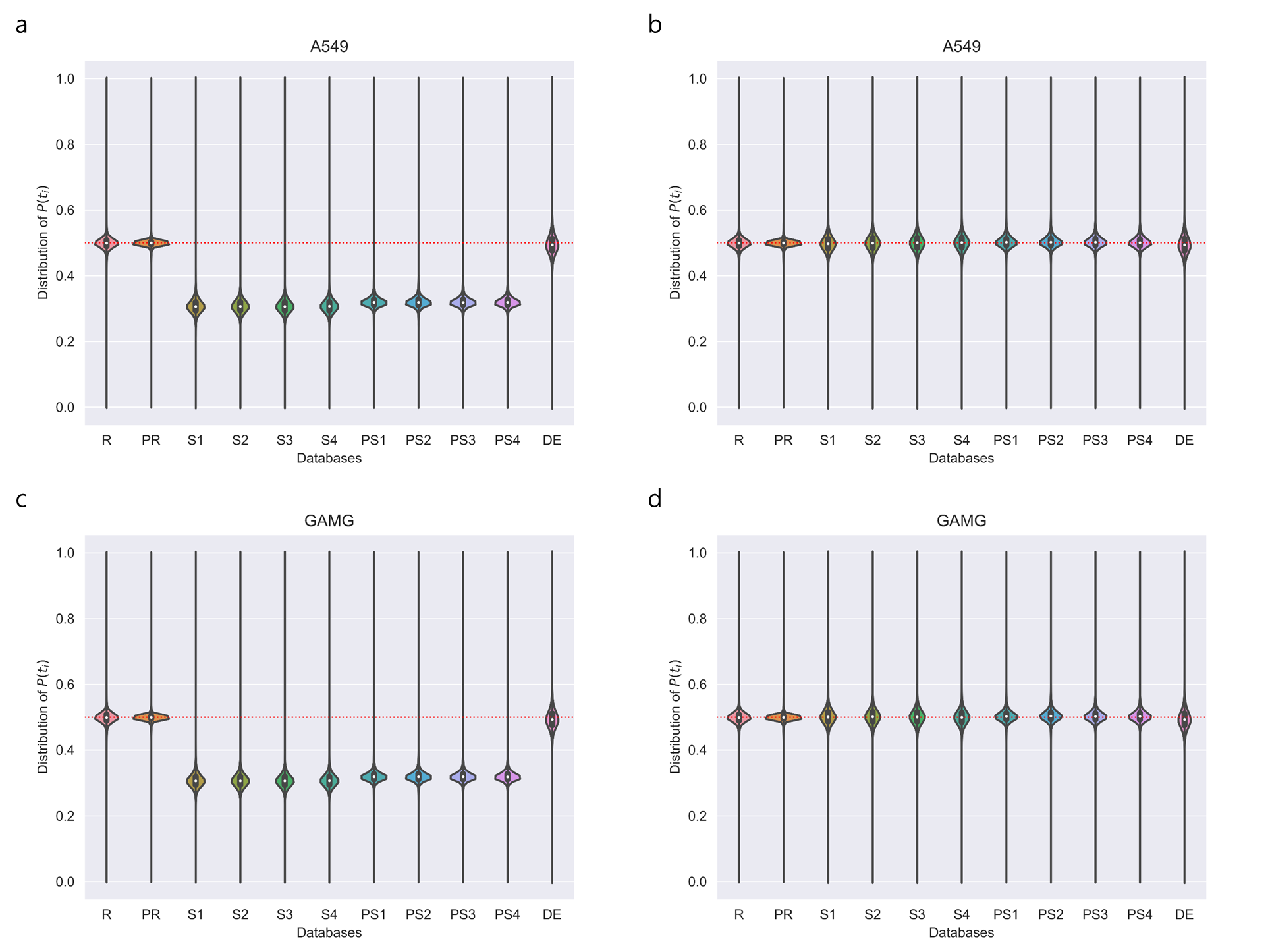
** **
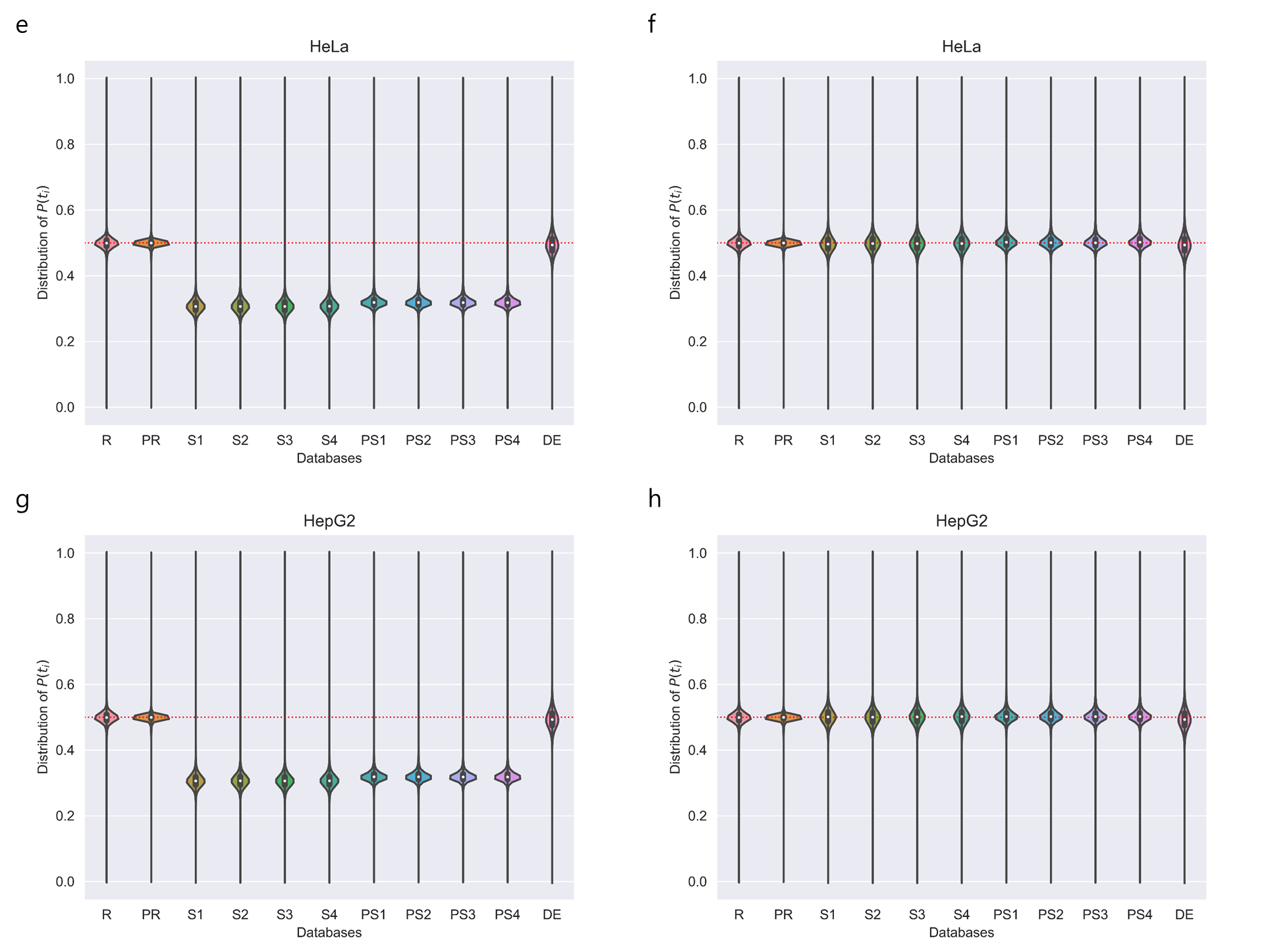
**

**
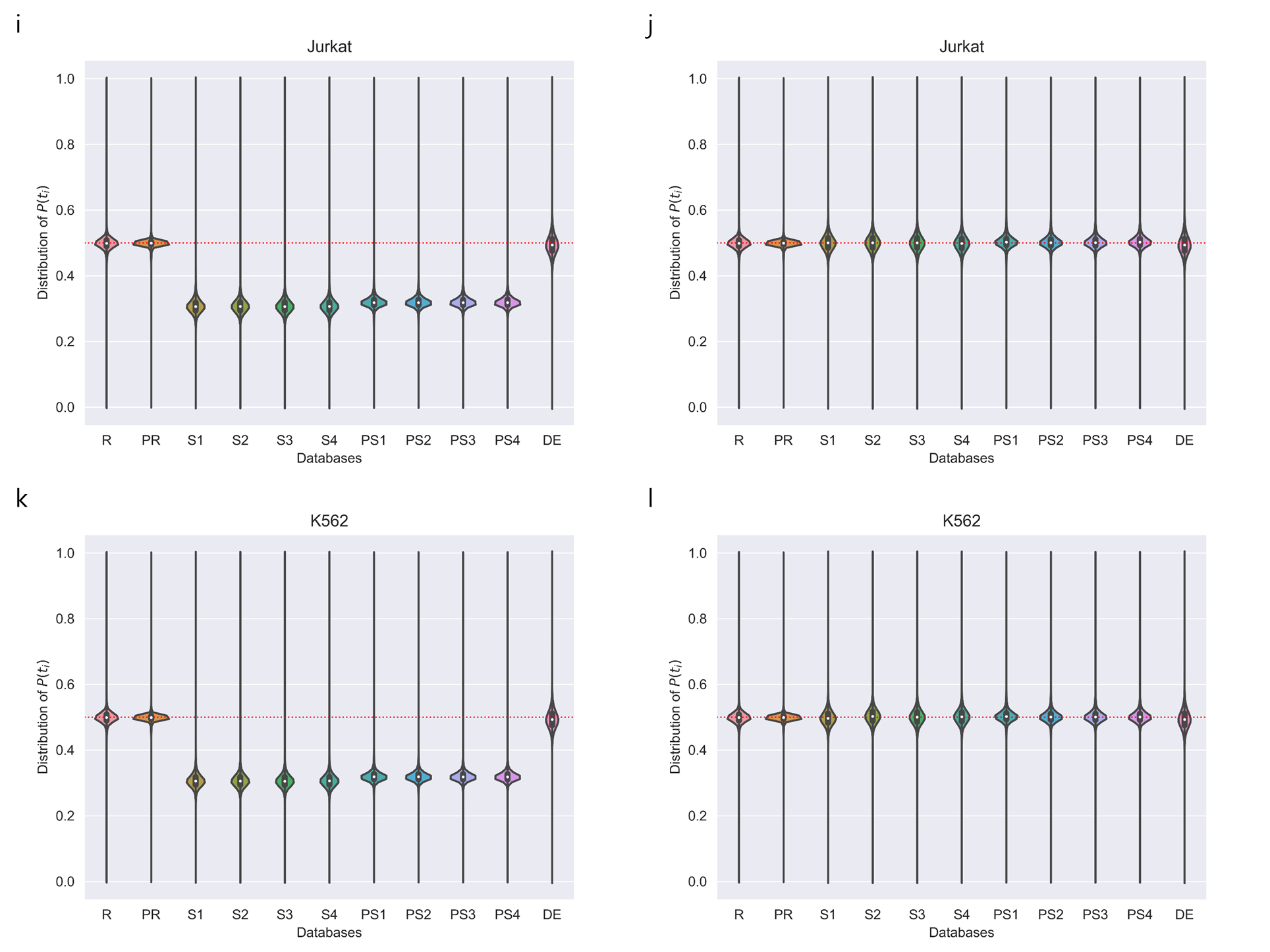
** **
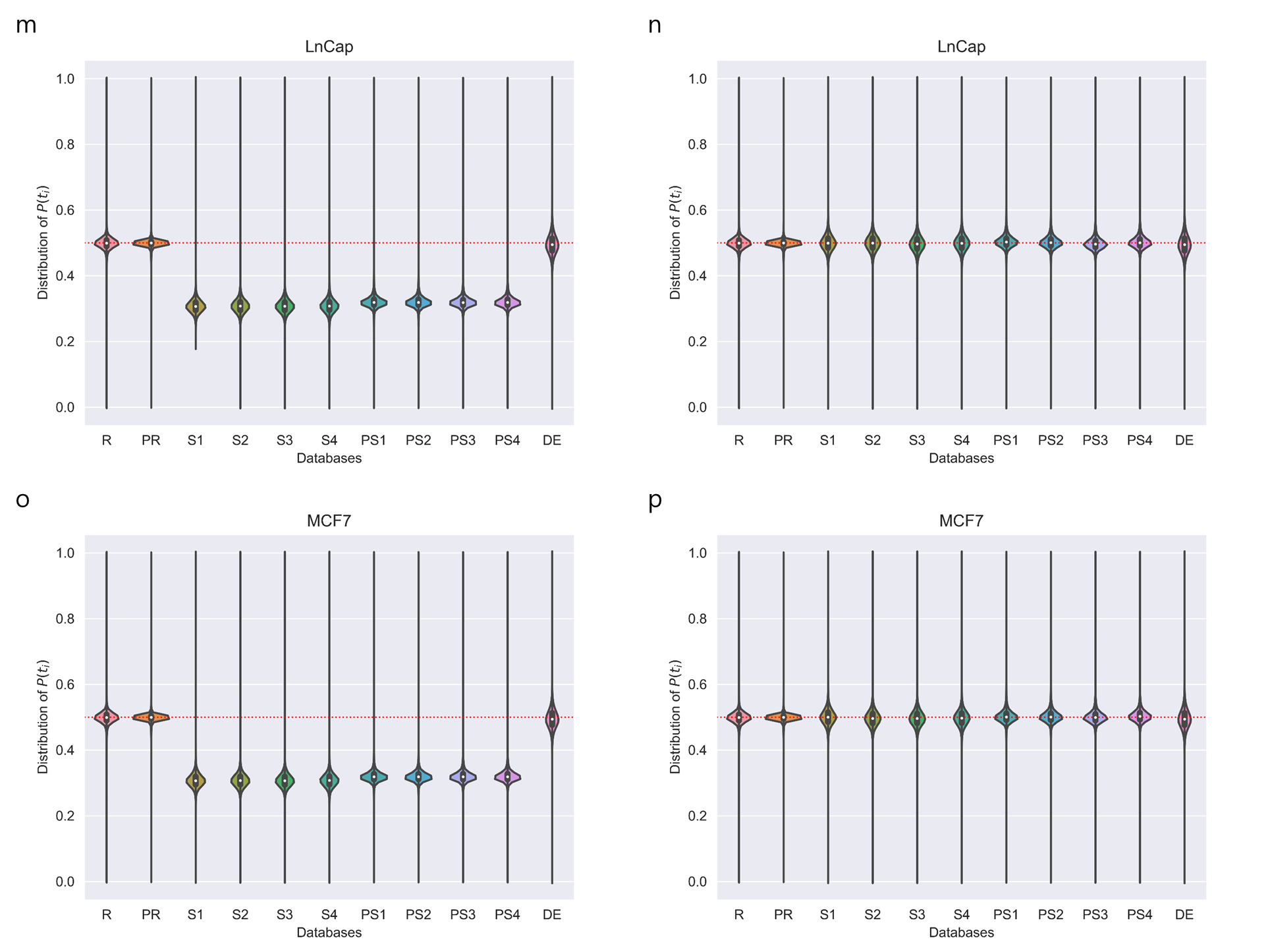
**

**
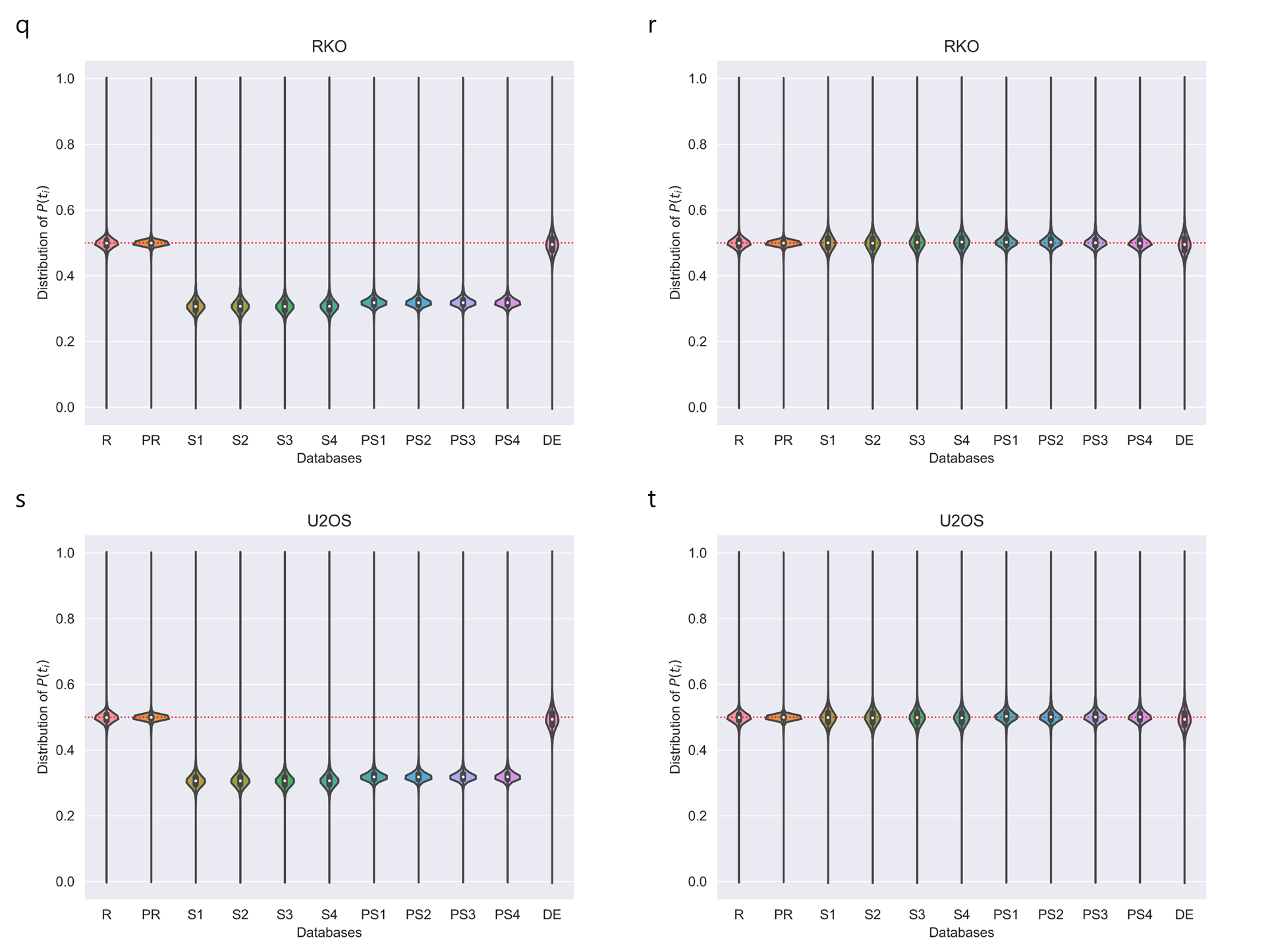
**

**Supplementary Figure 2.** Comparison of the distributions of $P(t_{i})$ of various databases and in the dataset. The x-axis represents the ratio of PSM with each $P(t_{i})$. The y-axis represents different databases. A549 (a, b), GAMG (c, d), HeLa (e, f), HepG2 (g, h), Jurkat (i, j), K562 (k, l), LnCap (m, n), MCF7 (o, p), RKO (q, r), U2OS (s, t). Each dataset is shown without a correction factor and with a correction factor. For example, A549 (a, b): (a) A549 without a correction factor, and (b) A549 with a correction factor.


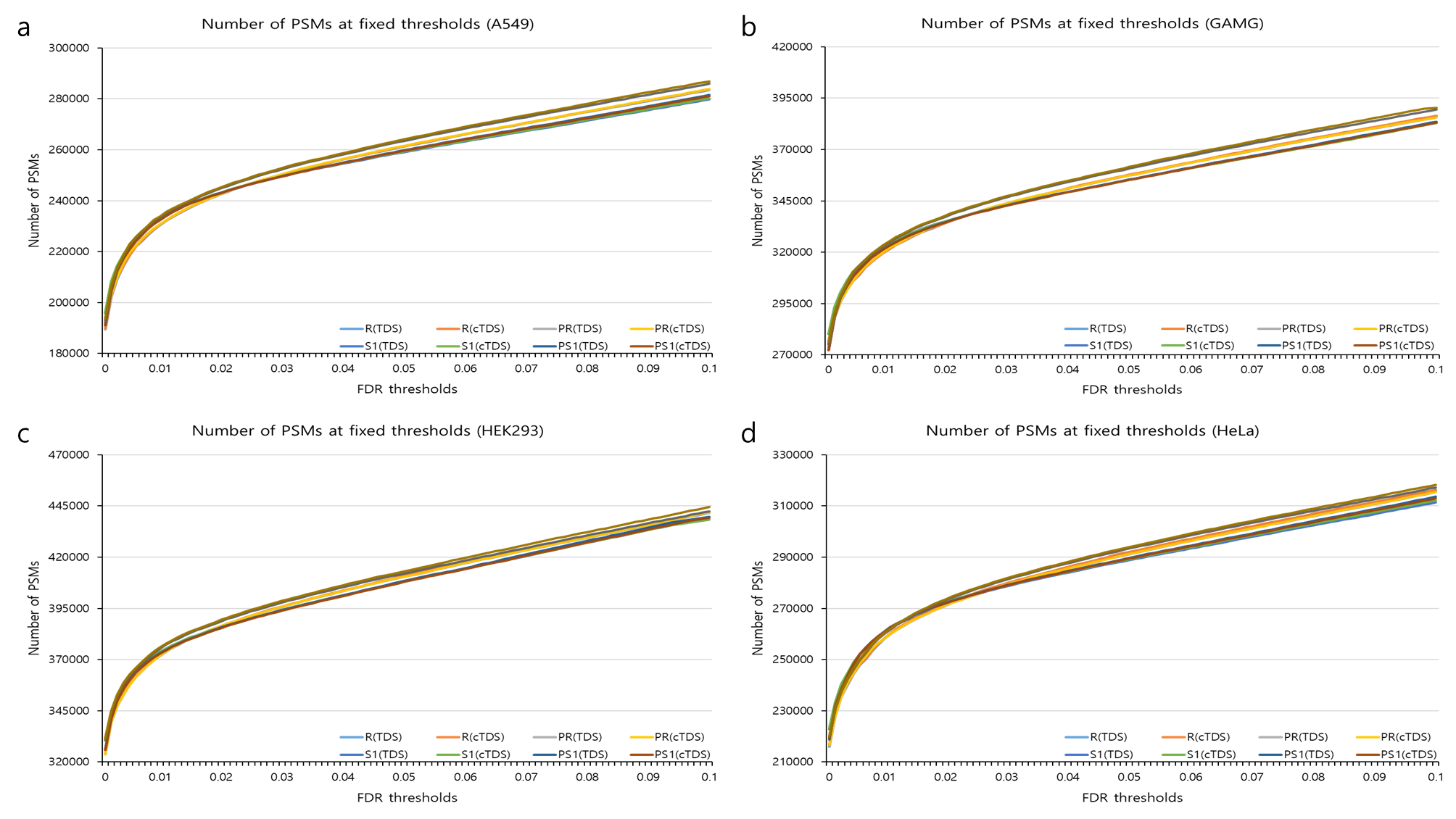


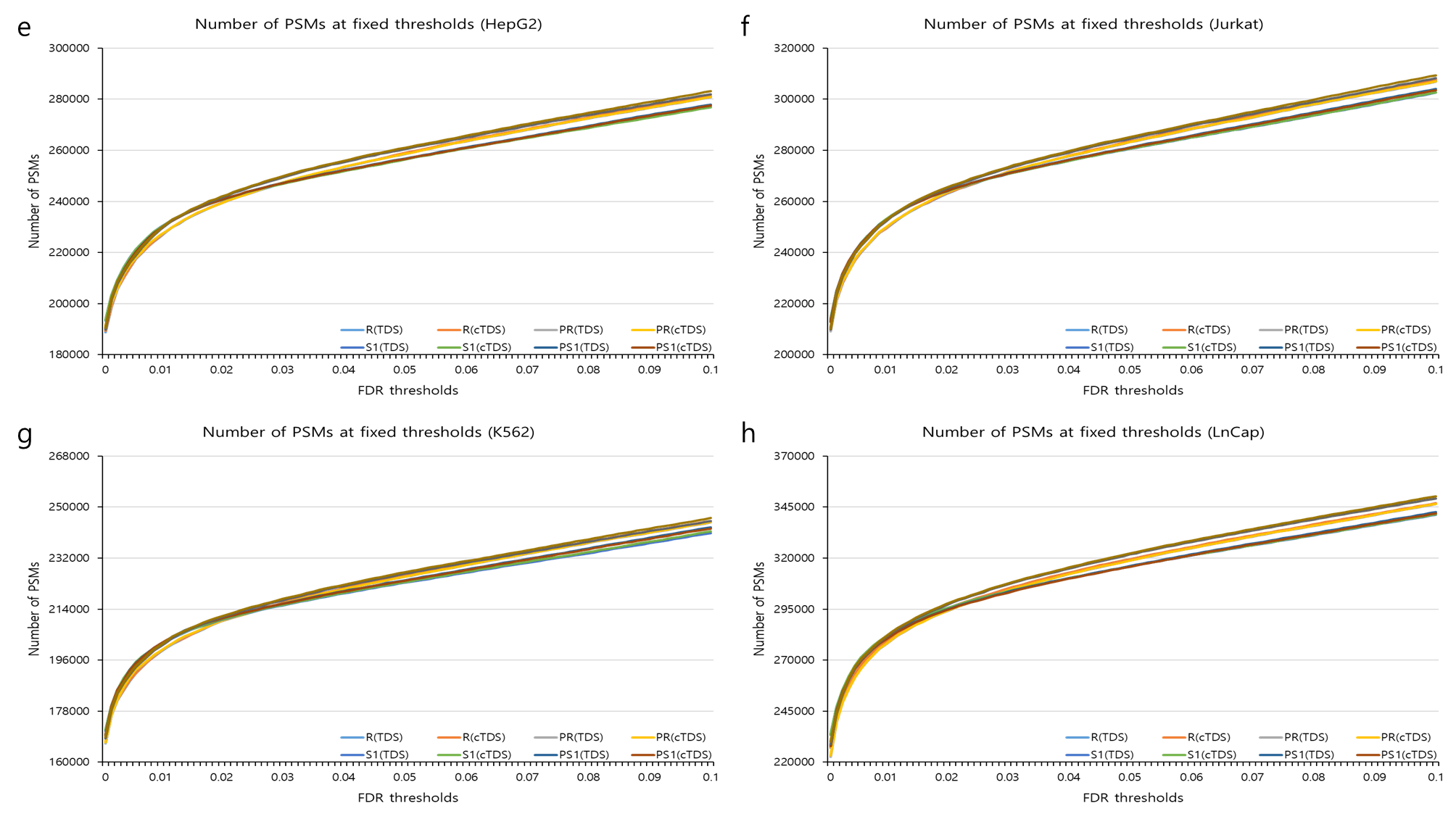


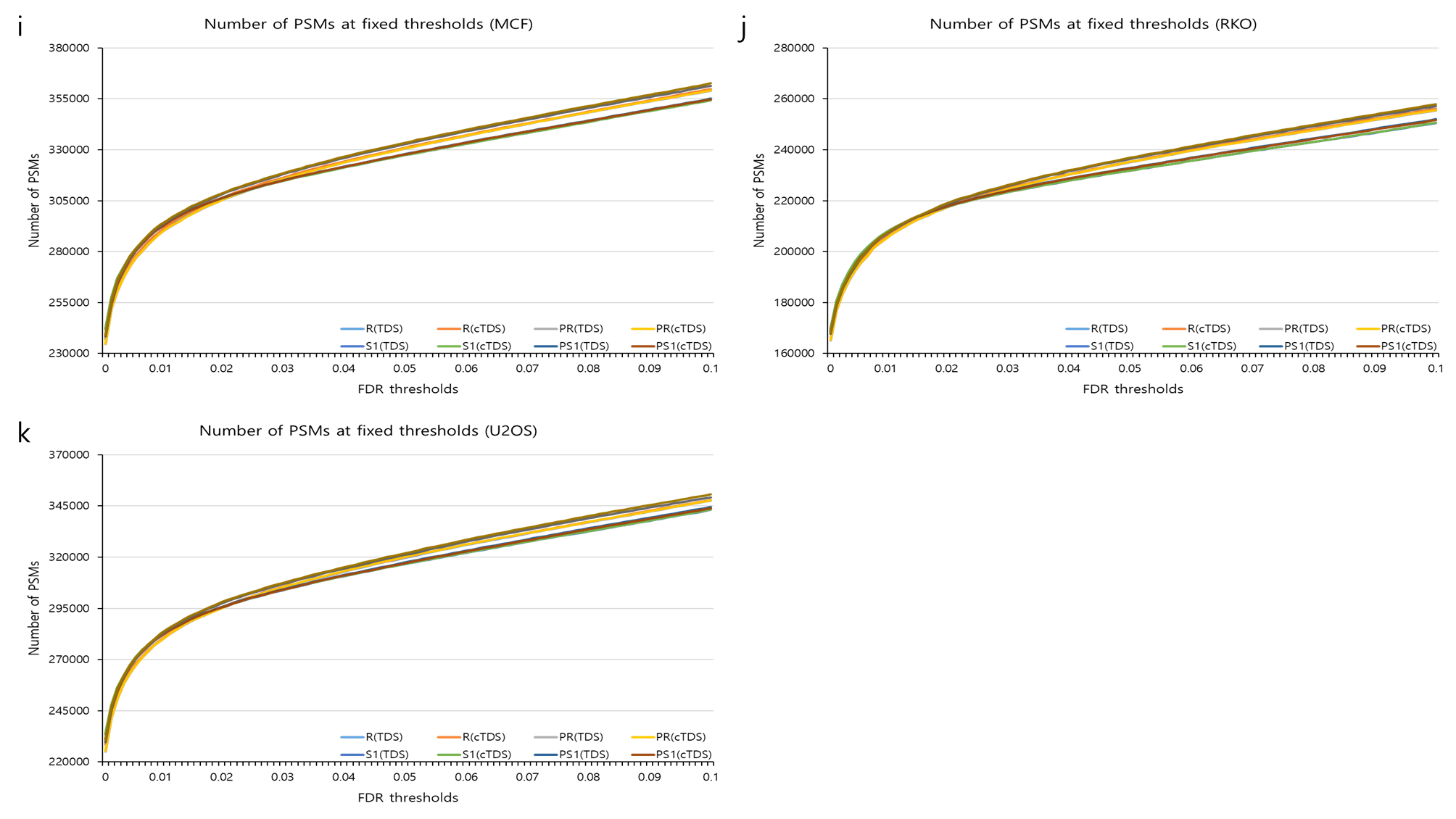


**Supplementary Figure 3.** Comparison of the number of PSMs in various databases and at a fixed FDR threshold. Each line corresponds to the number of PSMs estimated by TDS and cTDS at fixed FDR thresholds. (a) A549, (b) GAMG, (c) HEK293, (d) HeLa, (e) HepG2, (f) Jurkat, (g) K562, (h) LnCap, (i) MCF7, (j) RKO, and (k) U2OS.

**
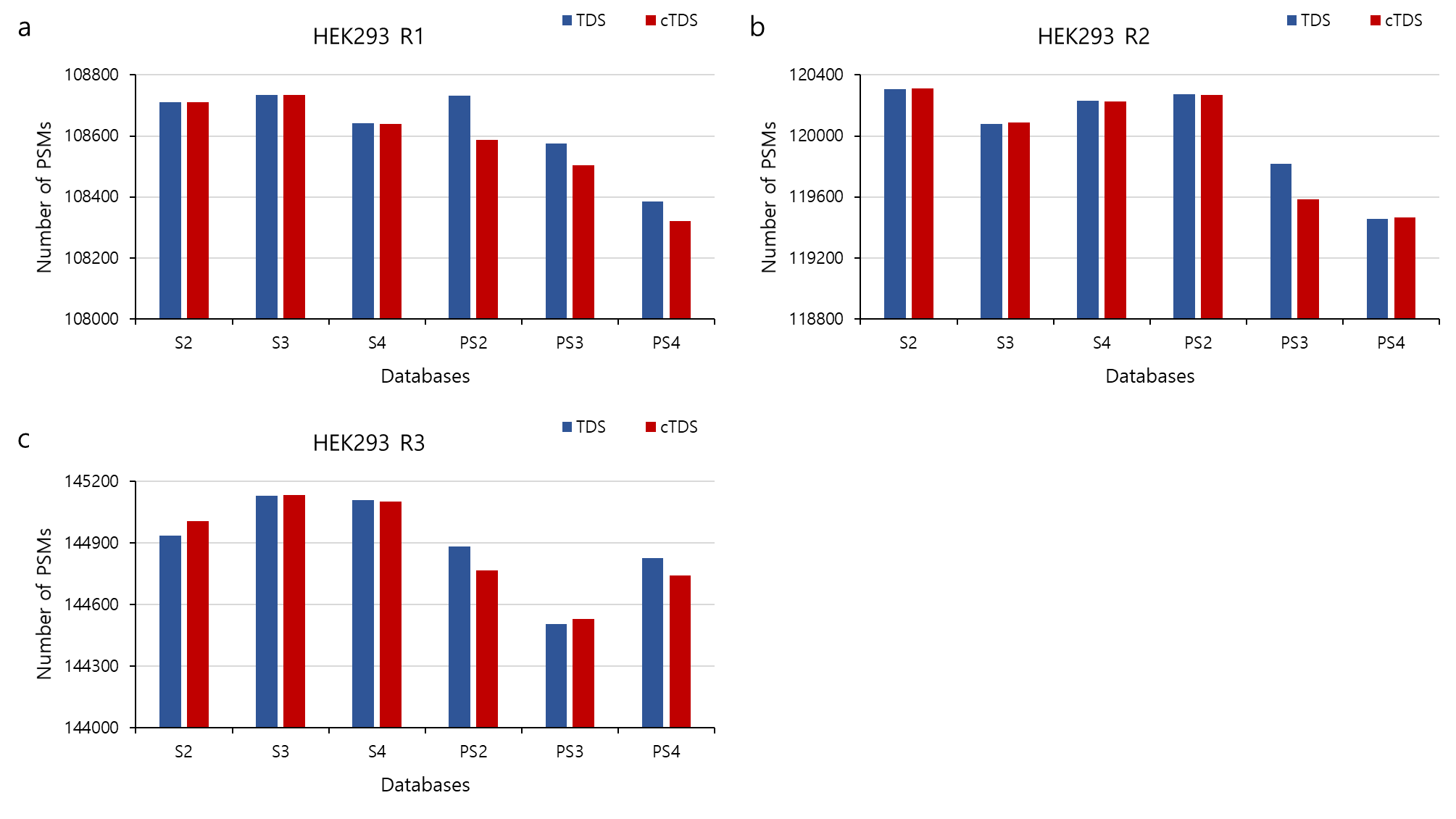
**

**Supplementary Figure 4.** Comparison of the number of PSMs of the stochastic databases. The blue bar shows the number of PSMs obtained with TDS at the 1% FDR threshold. The red bar shows the number of PSMs obtained with cTDS at the 1% FDR threshold: (a) HEK293 first replicate, (b) HEK293 second replicate, and (c) HEK293 third replicate.

**
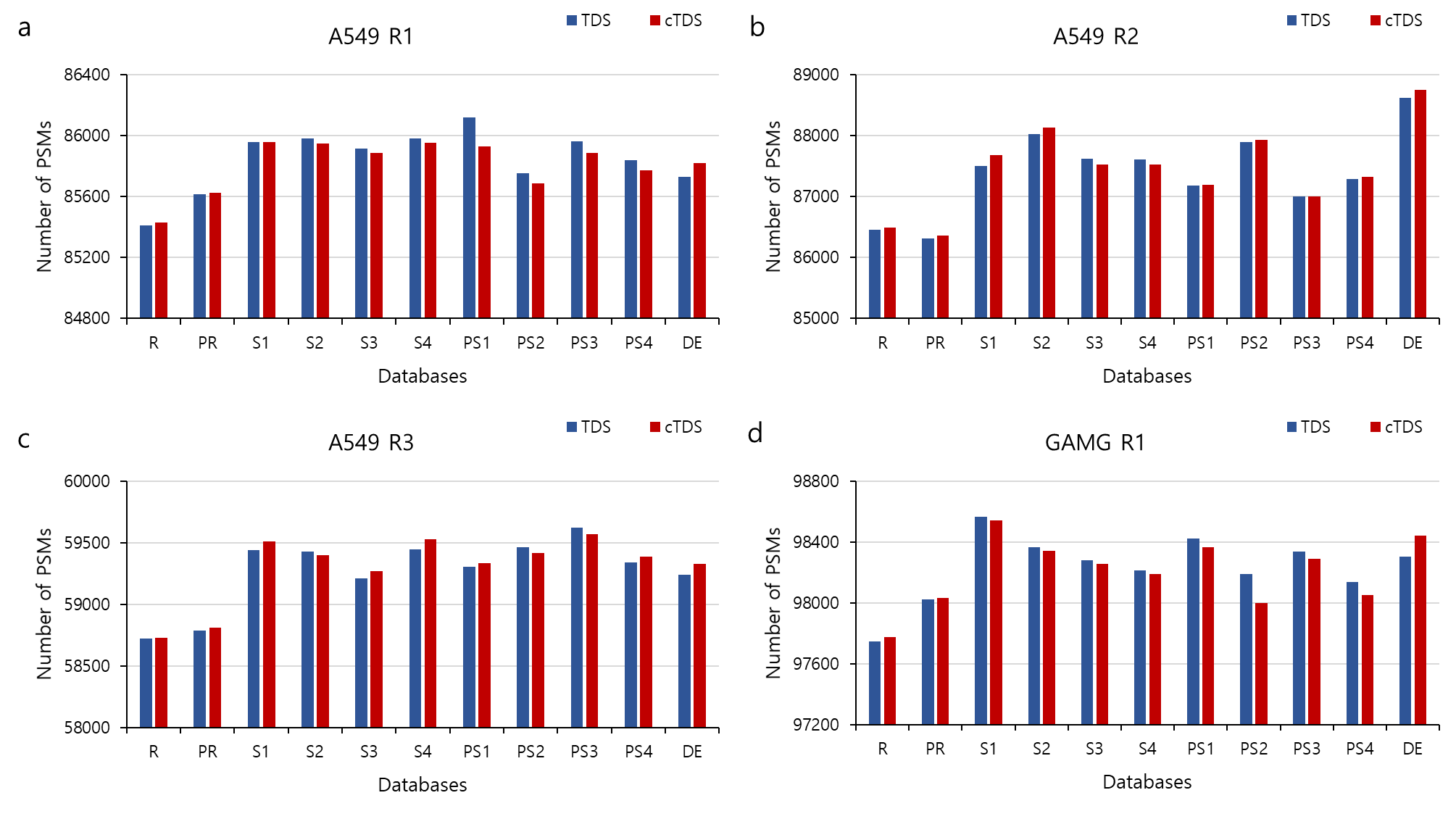
** **
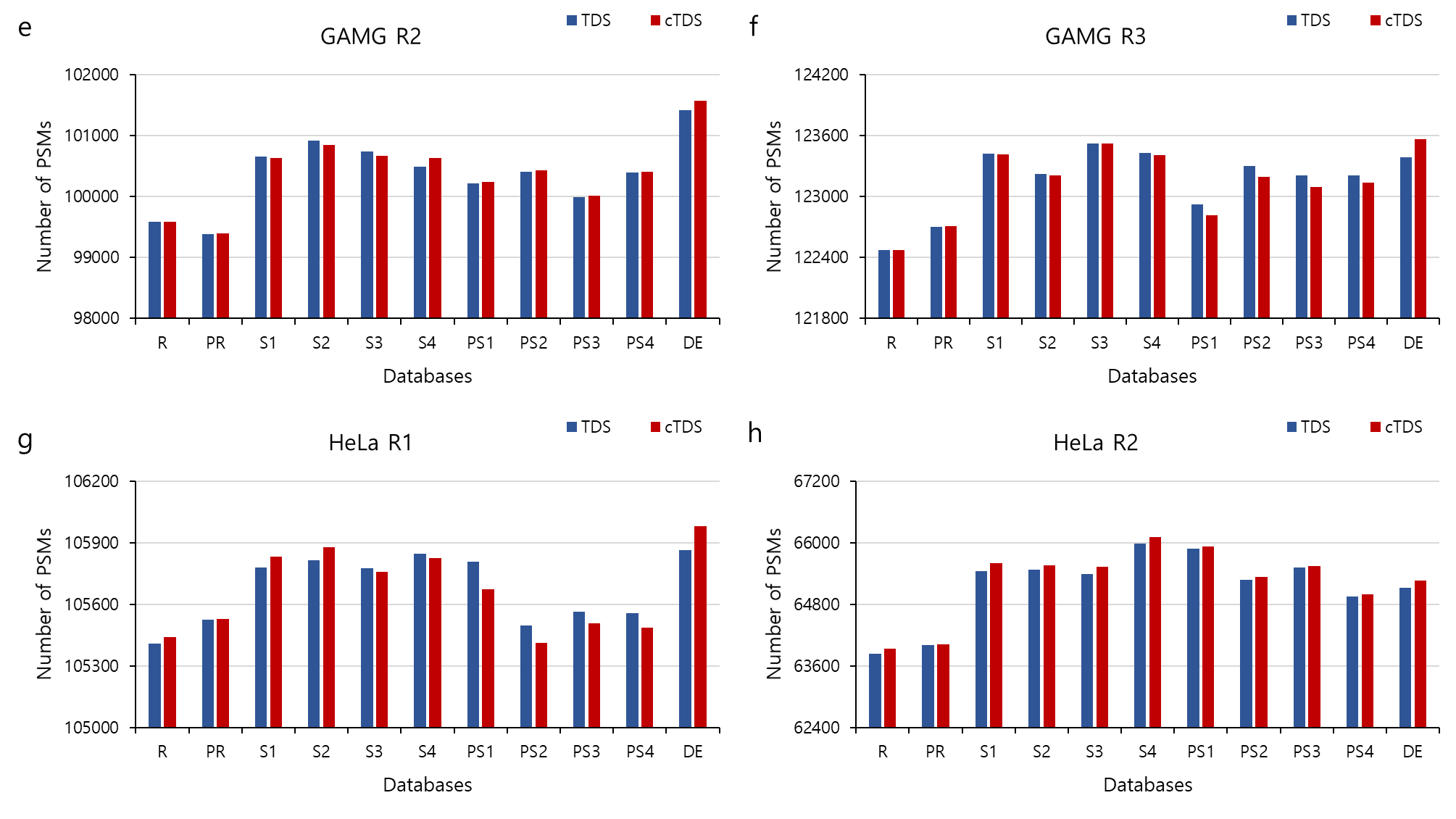
**
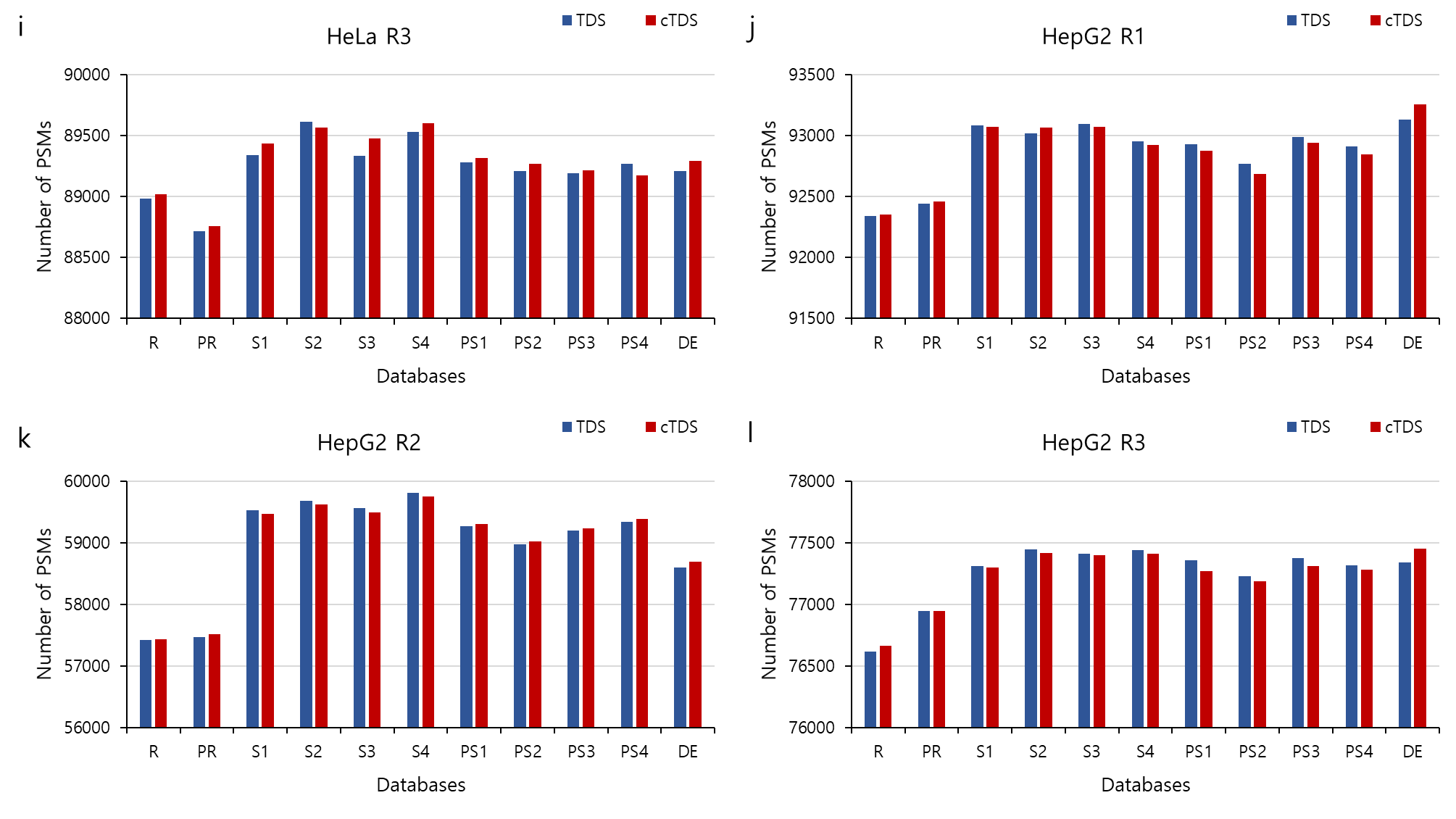

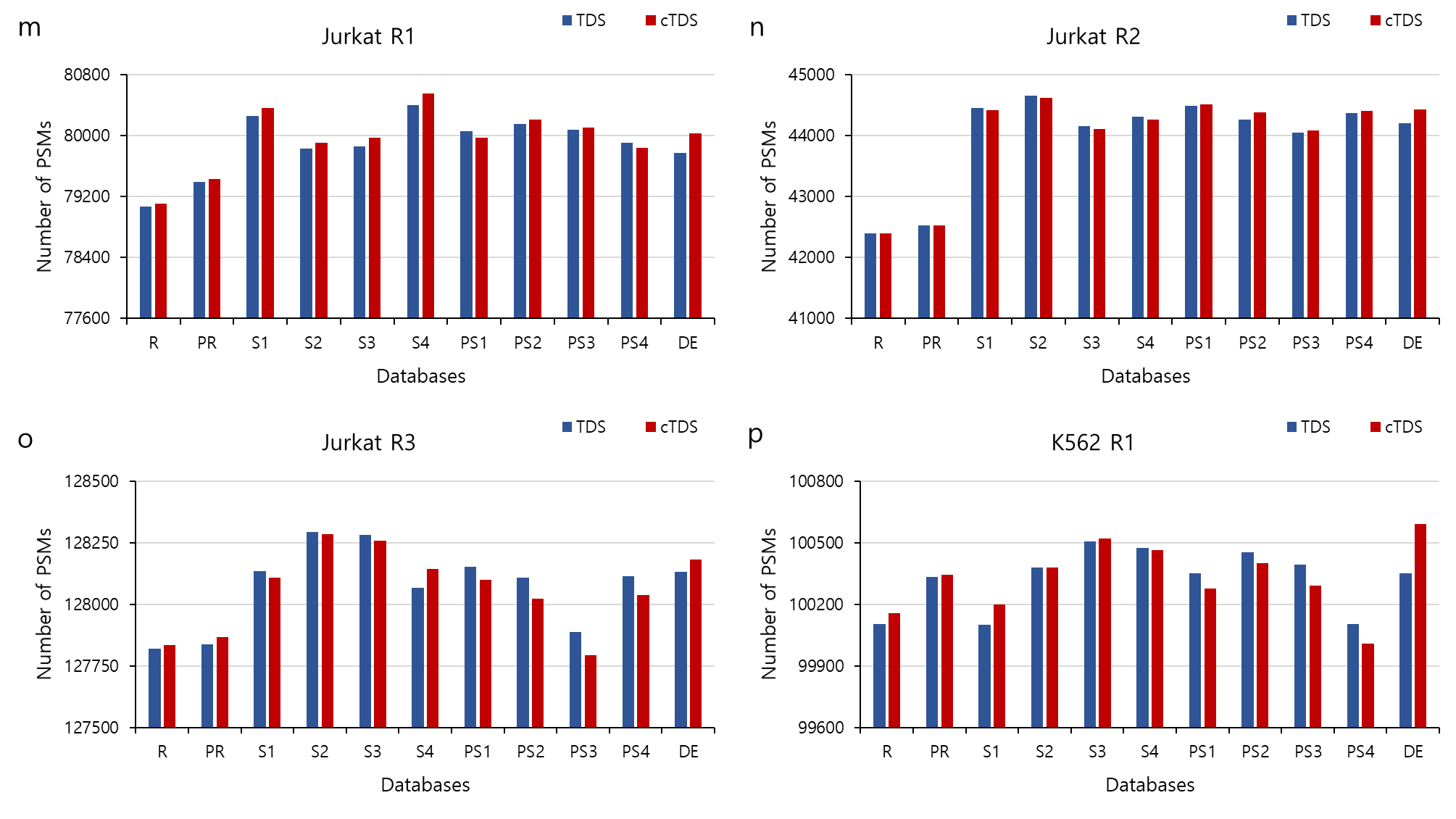

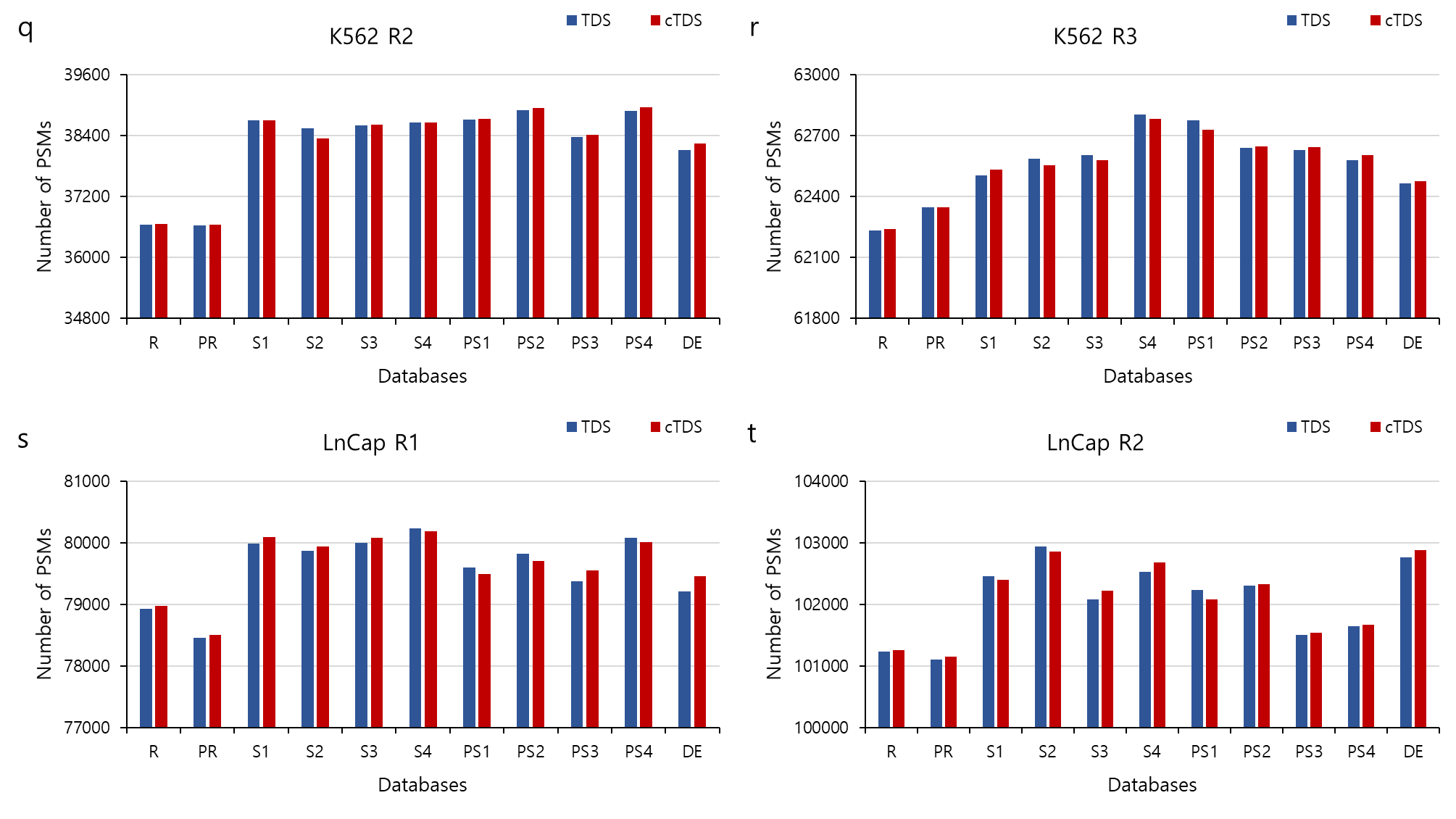

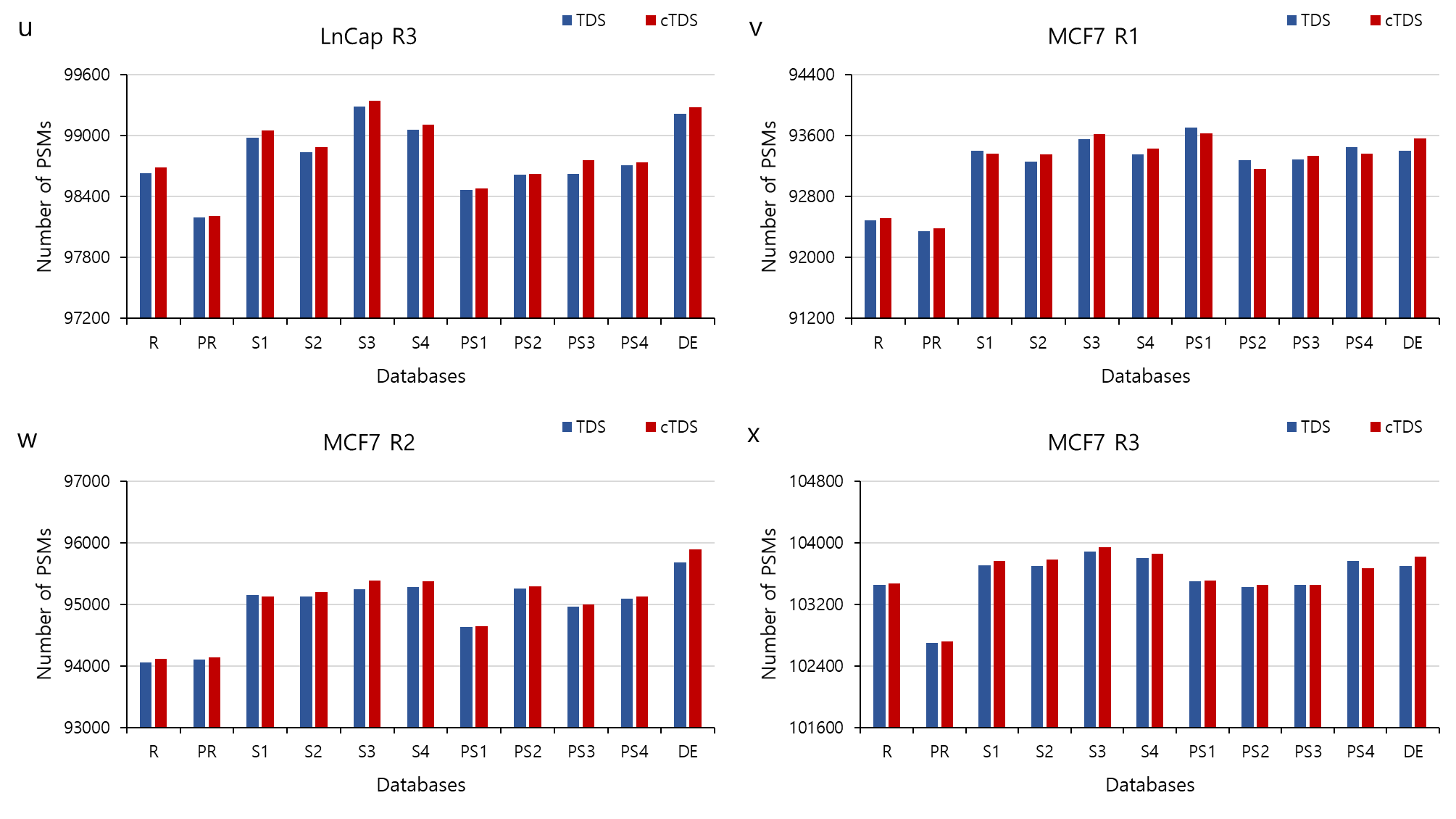

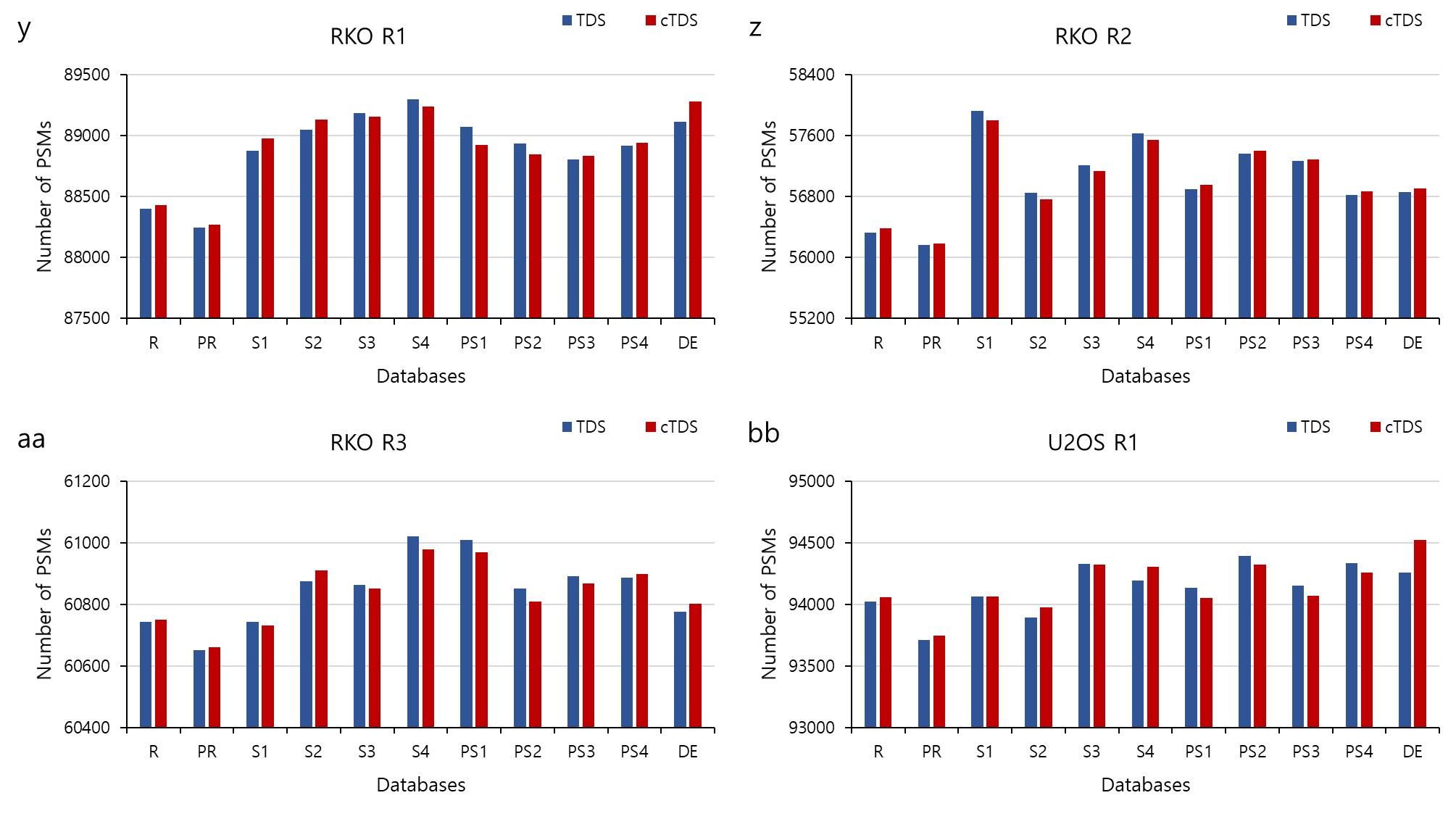

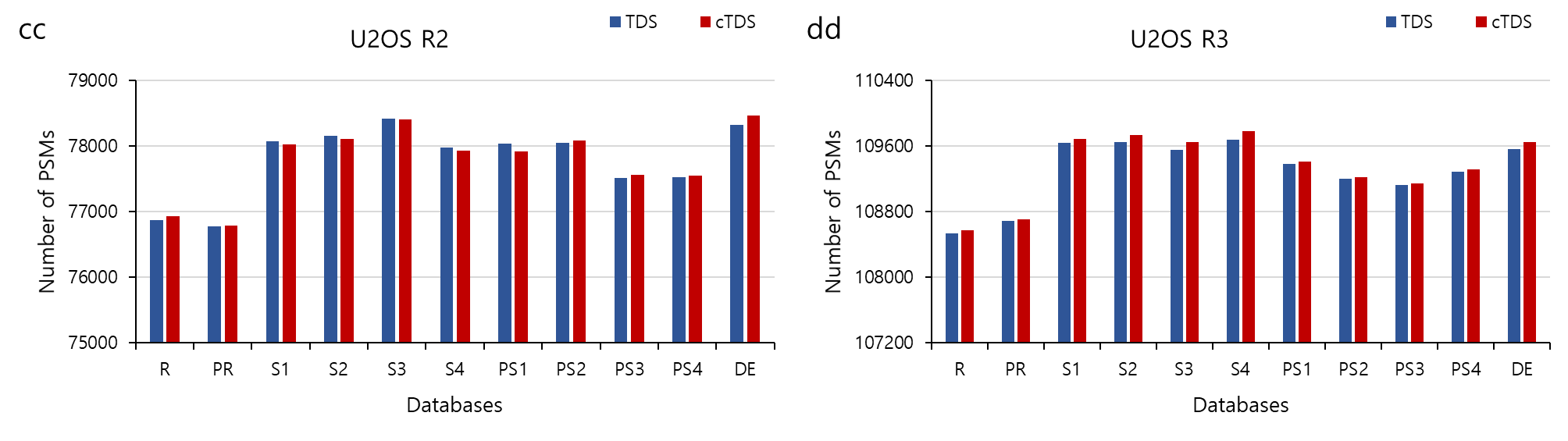


**Supplementary Figure 5.** Comparison of the number of PSMs of various databases and datasets. The blue bar shows the number of PSMs obtained with TDS at the 1% FDR threshold. The red bar shows the number of PSMs obtained with cTDS at the 1% FDR threshold. A549 (a-c), GAMG (d-f), HeLa (g-i), HepG2 (j-l), Jurkat (m-o), K562 (p-r), LnCap (s-u), MCF7 (v-x), RKO (y-aa), U2OS (bb-dd). Each dataset is shown with the first replicate, second replicate, and third replicate. For example, A549 (a-c): (a) A549 first replicate, (b) A549 second replicate, and (c) A549 third replicate.

**
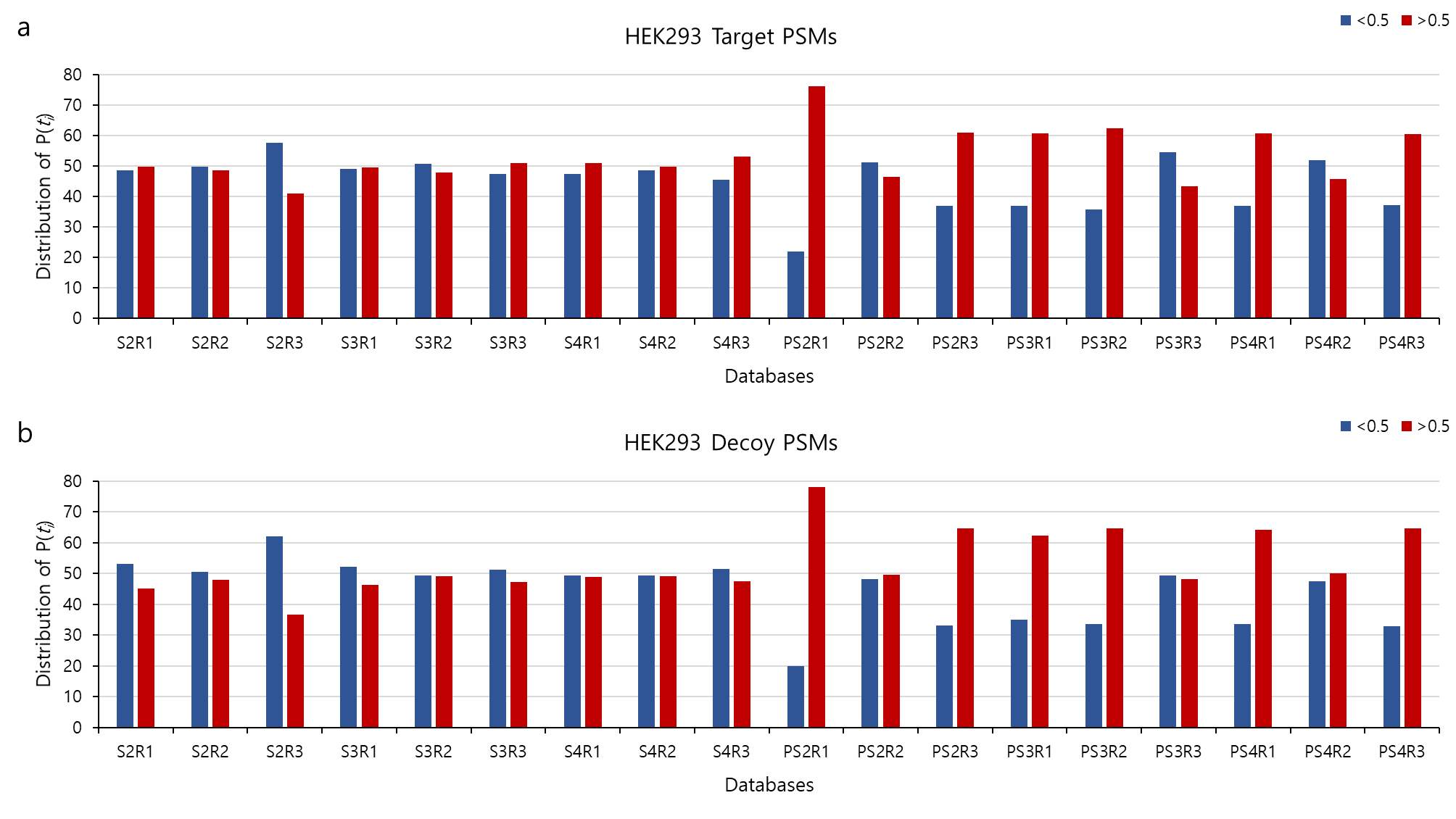
**

**Supplementary Figure 6.** Comparison of the $P(t_{i})$ distributions of the target and decoy hit rates for the stochastic databases and the HEK293 dataset. The blue bar shows the ratio of spectra for which $P(t_{i})$ < 0.5 among all spectra at a 1% FDR threshold. The red bar shows the ratio of spectra for which $P(t_{i})$ > 0.5 among all spectra at a 1% FDR threshold: (a) HEK293 target hits, and (b) HEK293 decoy hits.

**
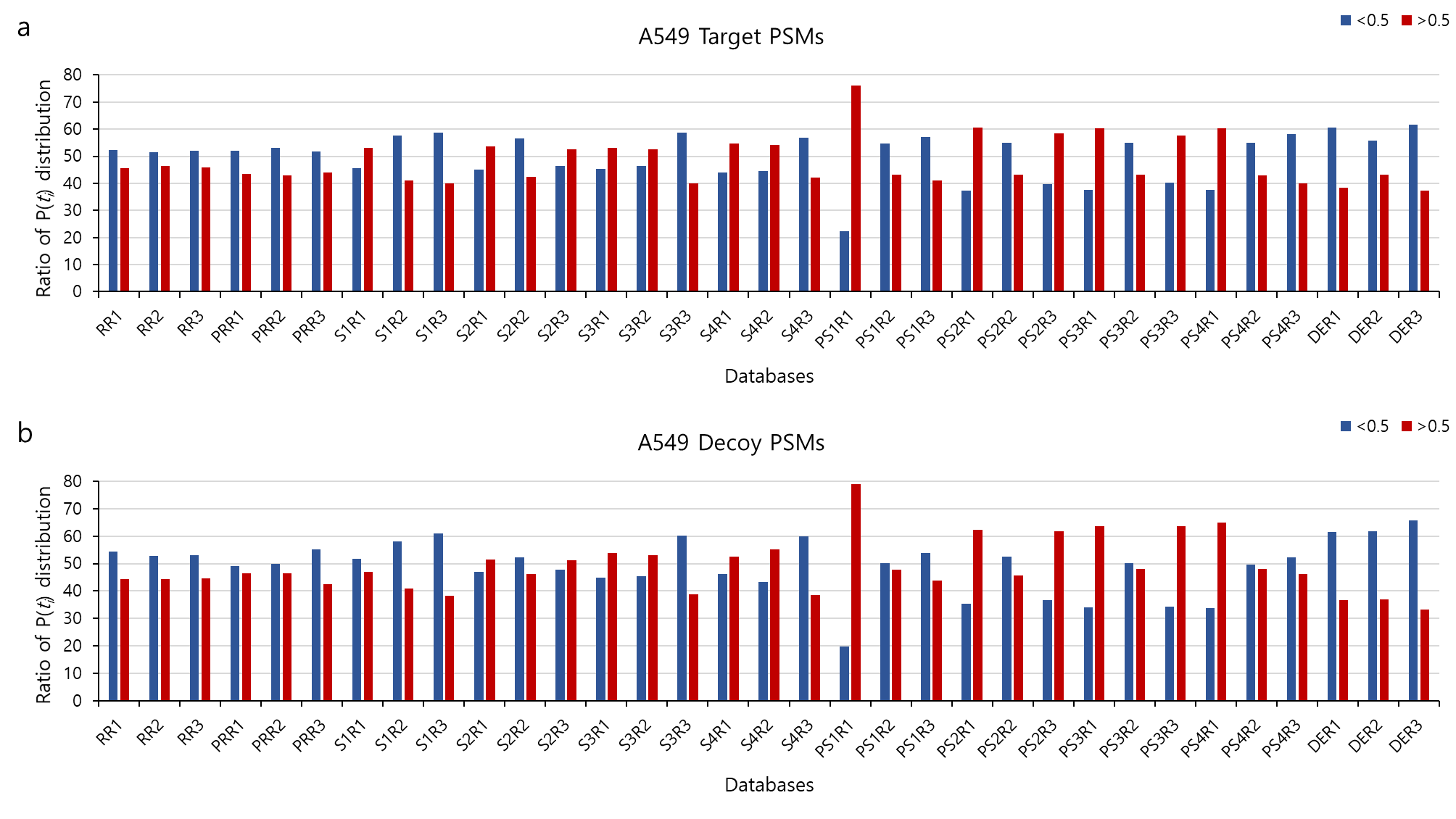
**

**
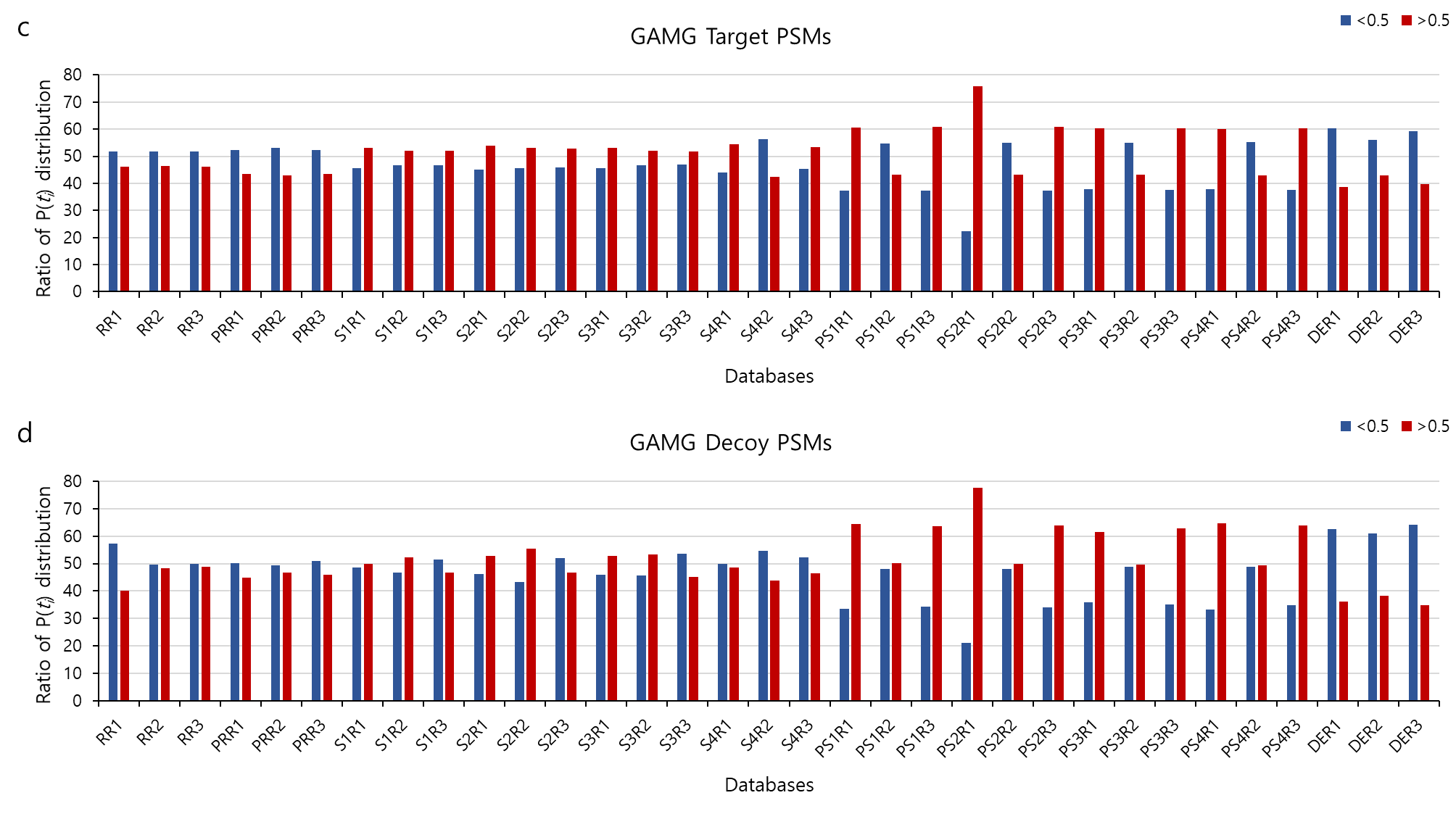
**

**
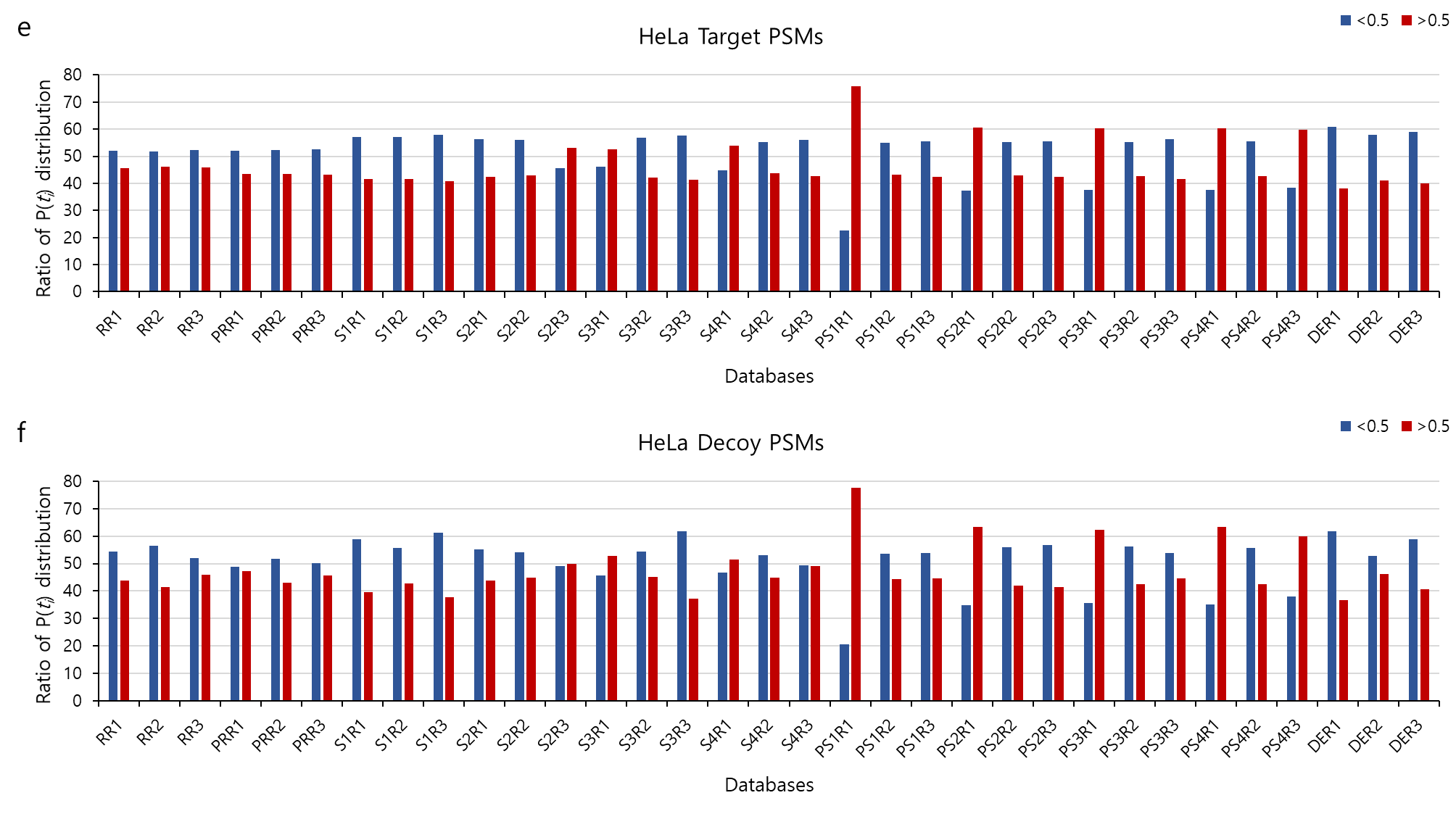
**

**
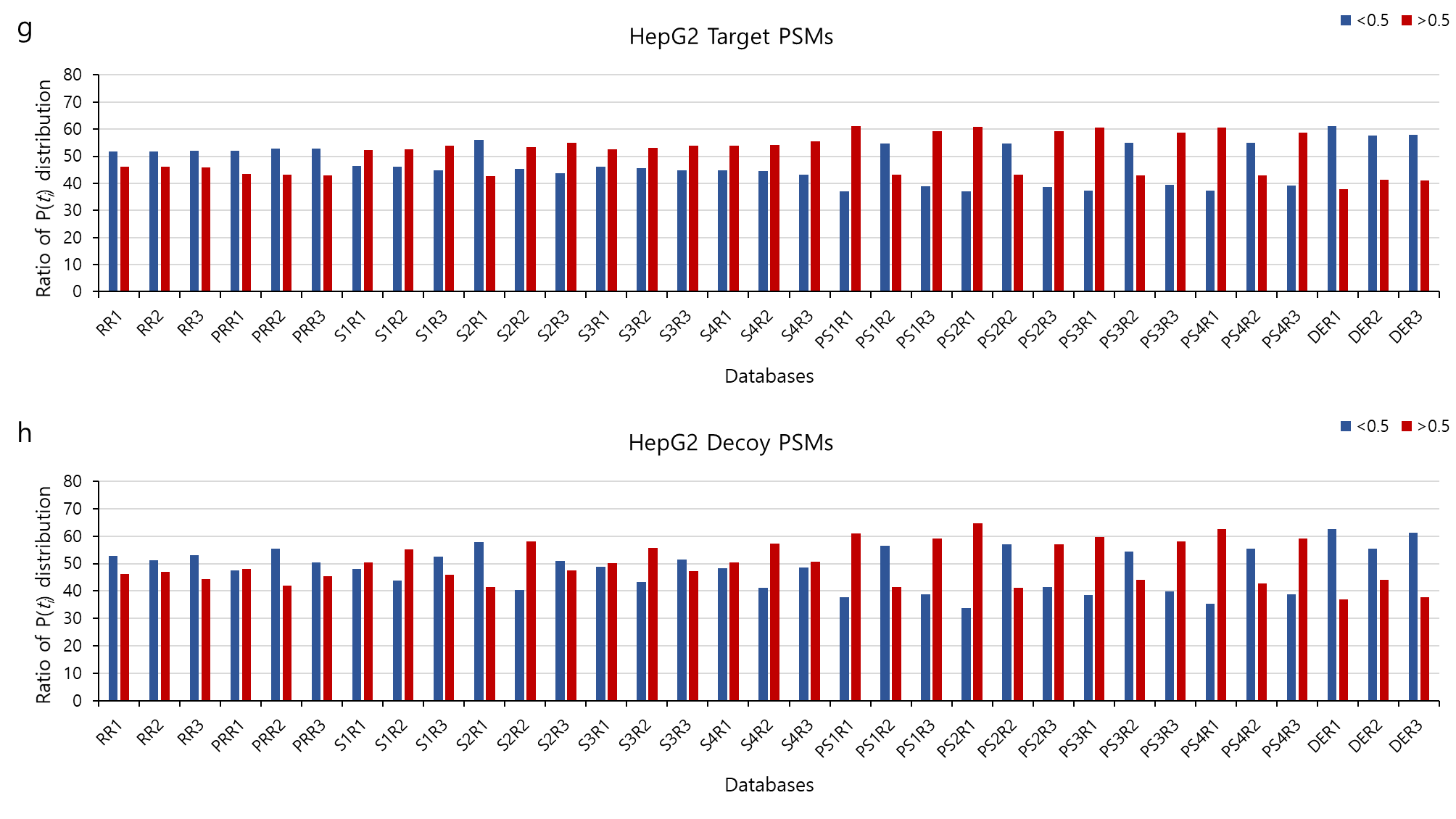
**

**
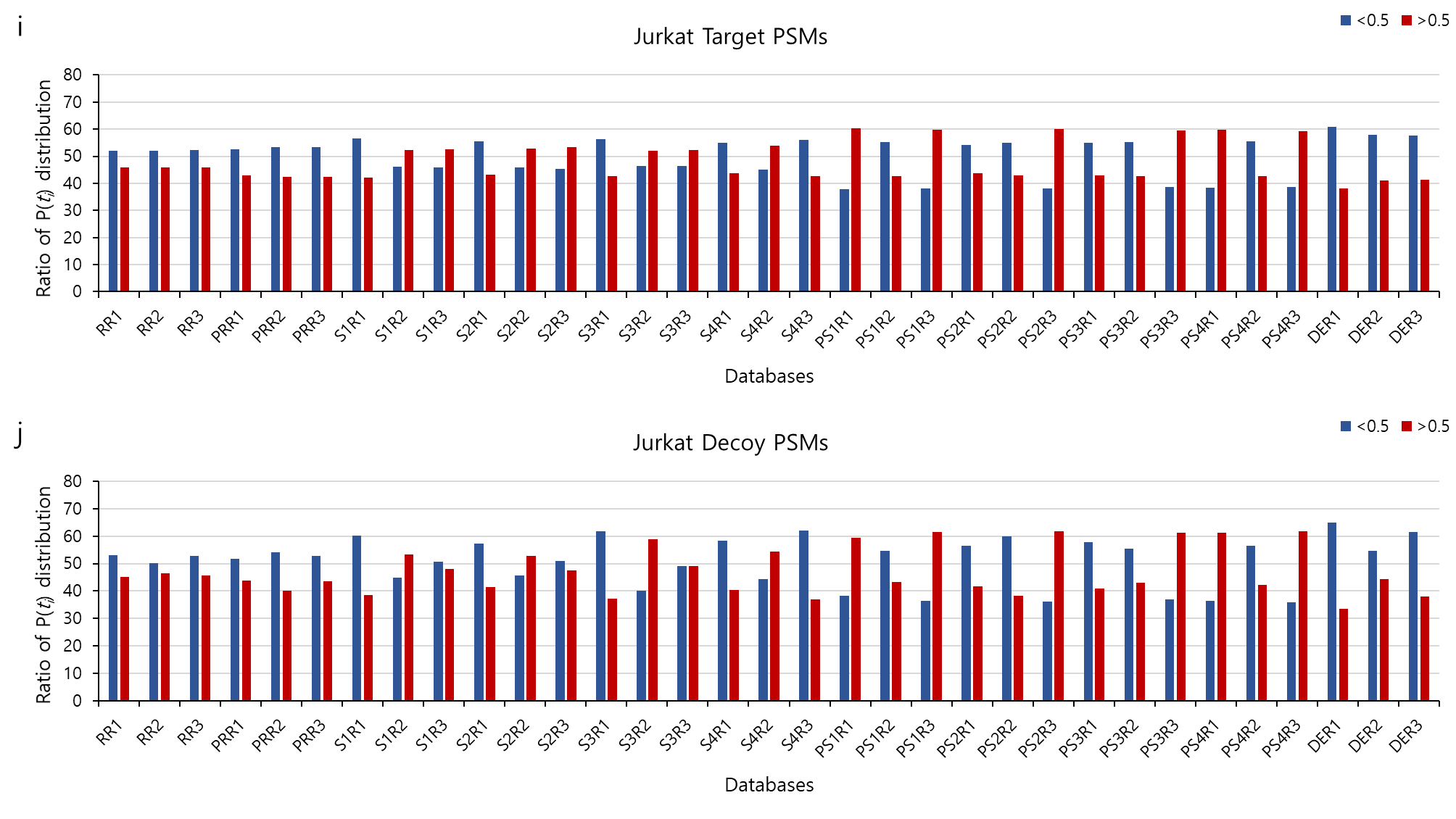
**

**
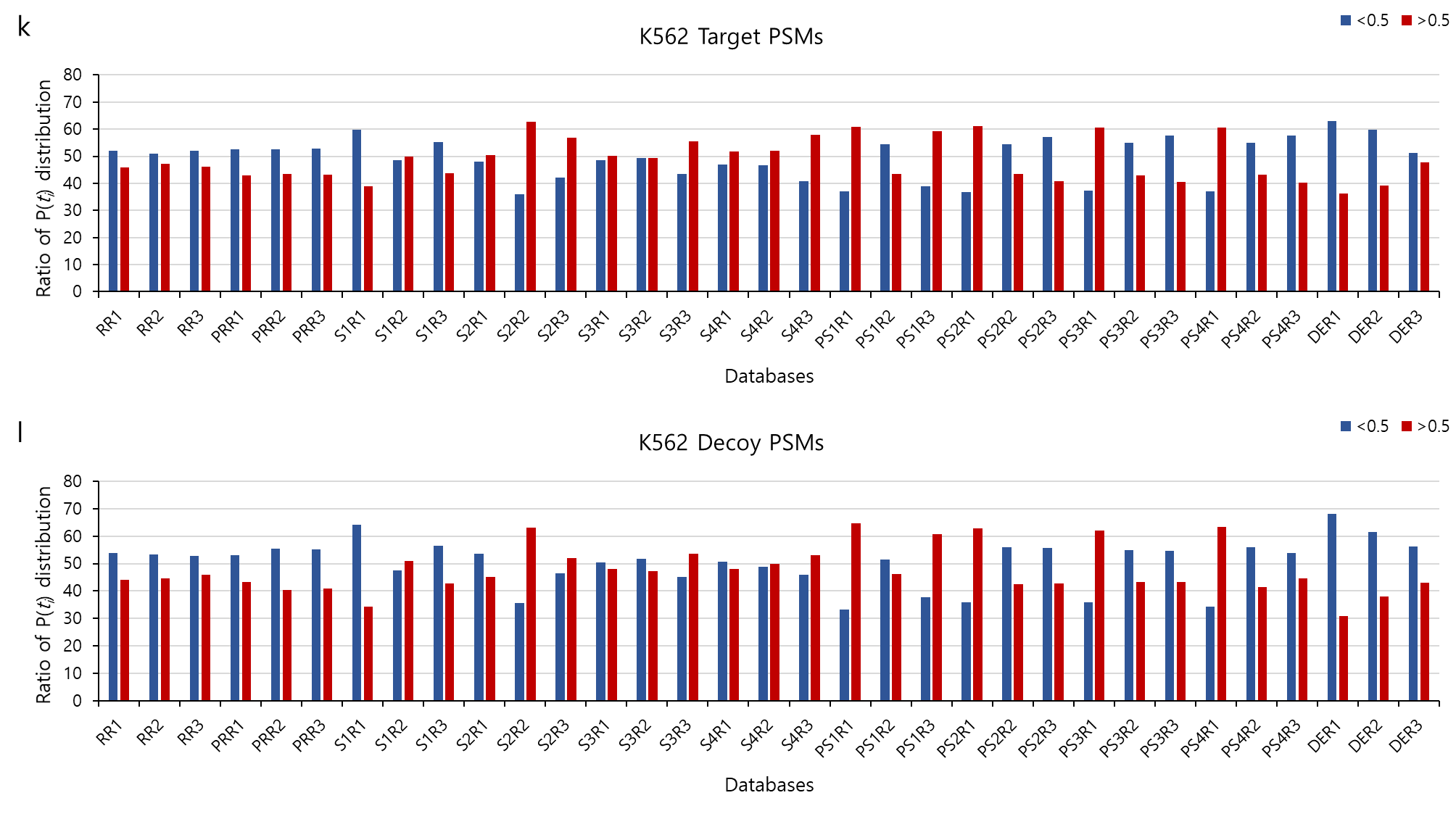
**

**
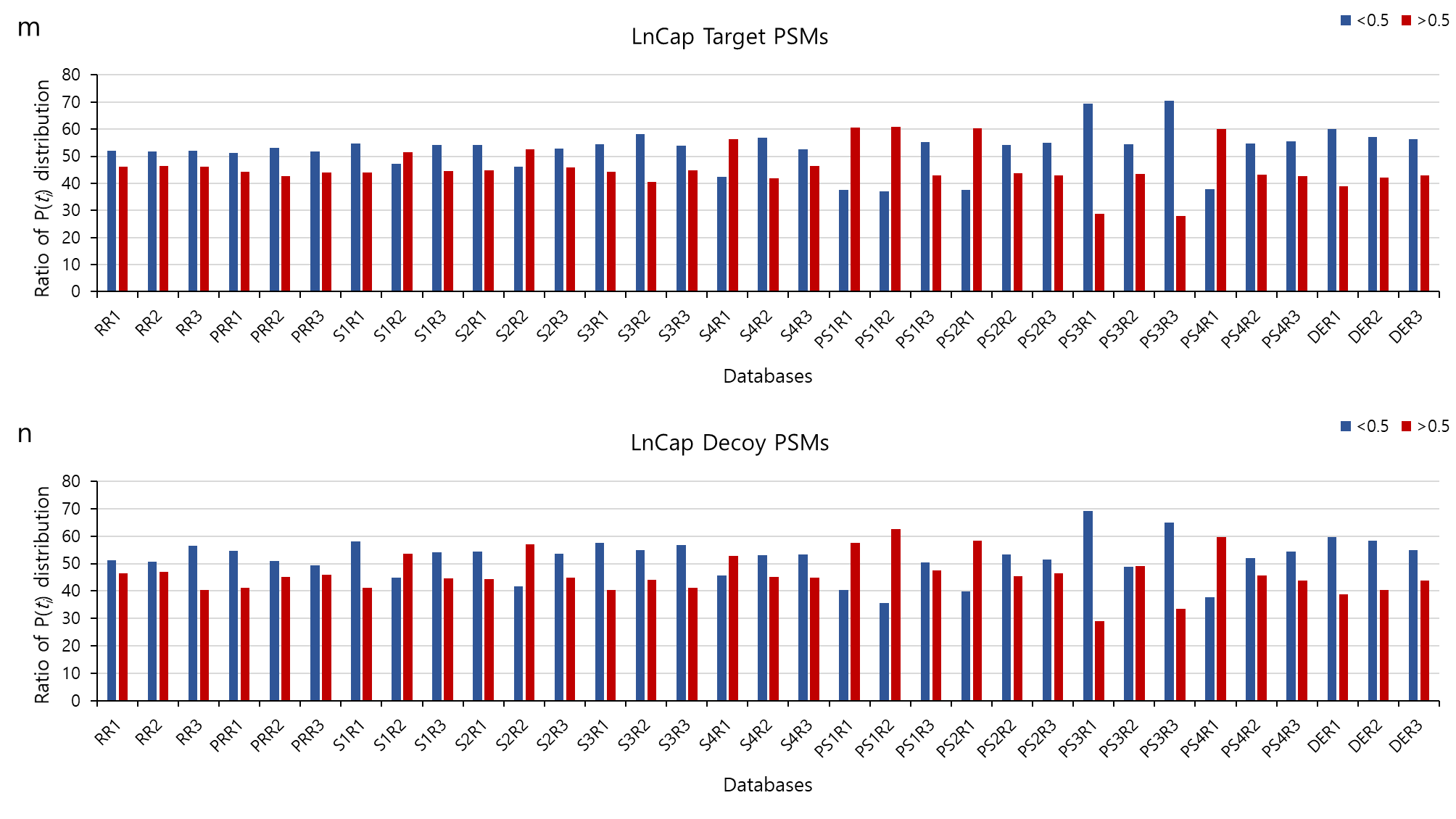
**

**
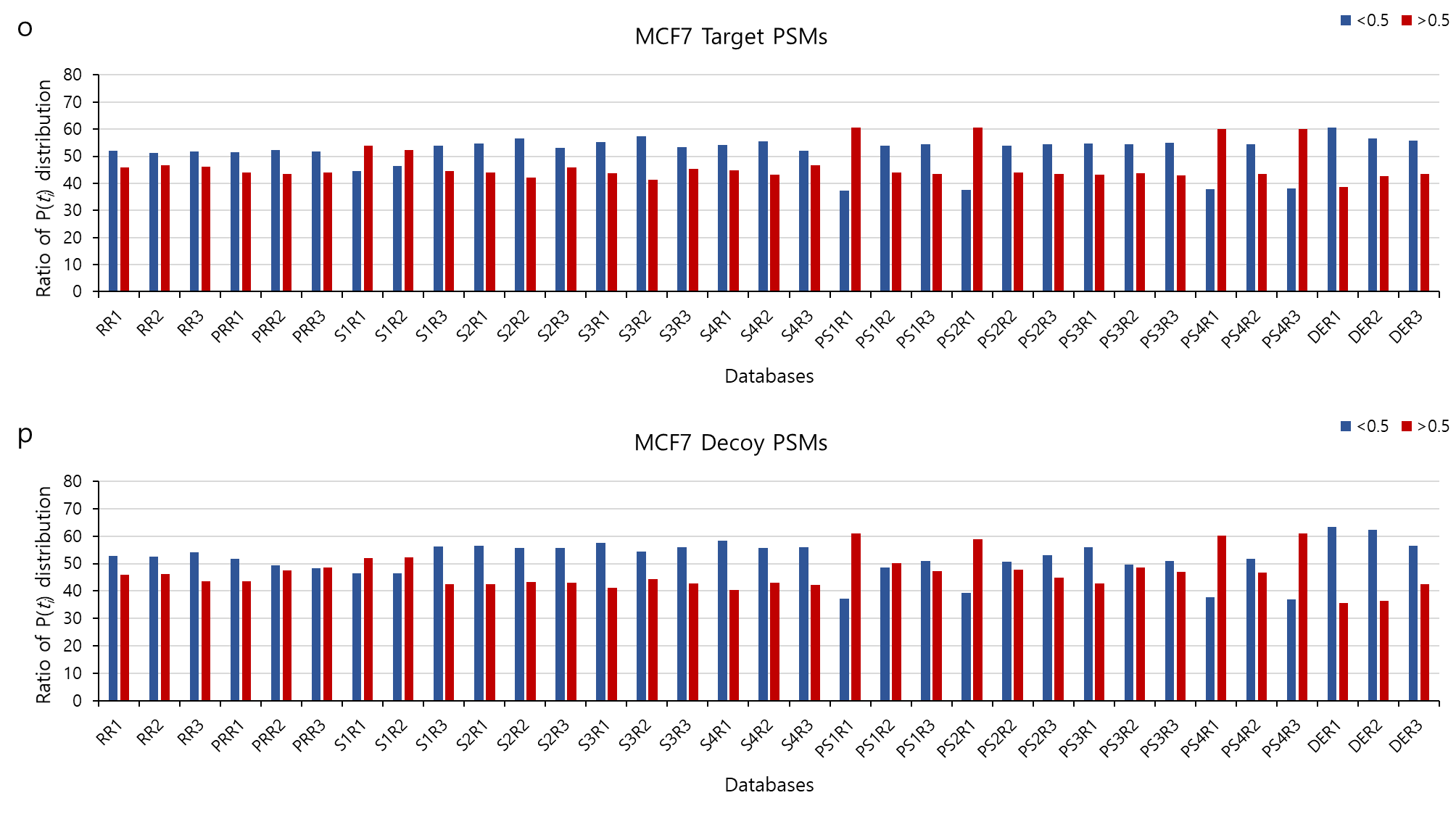
**

**
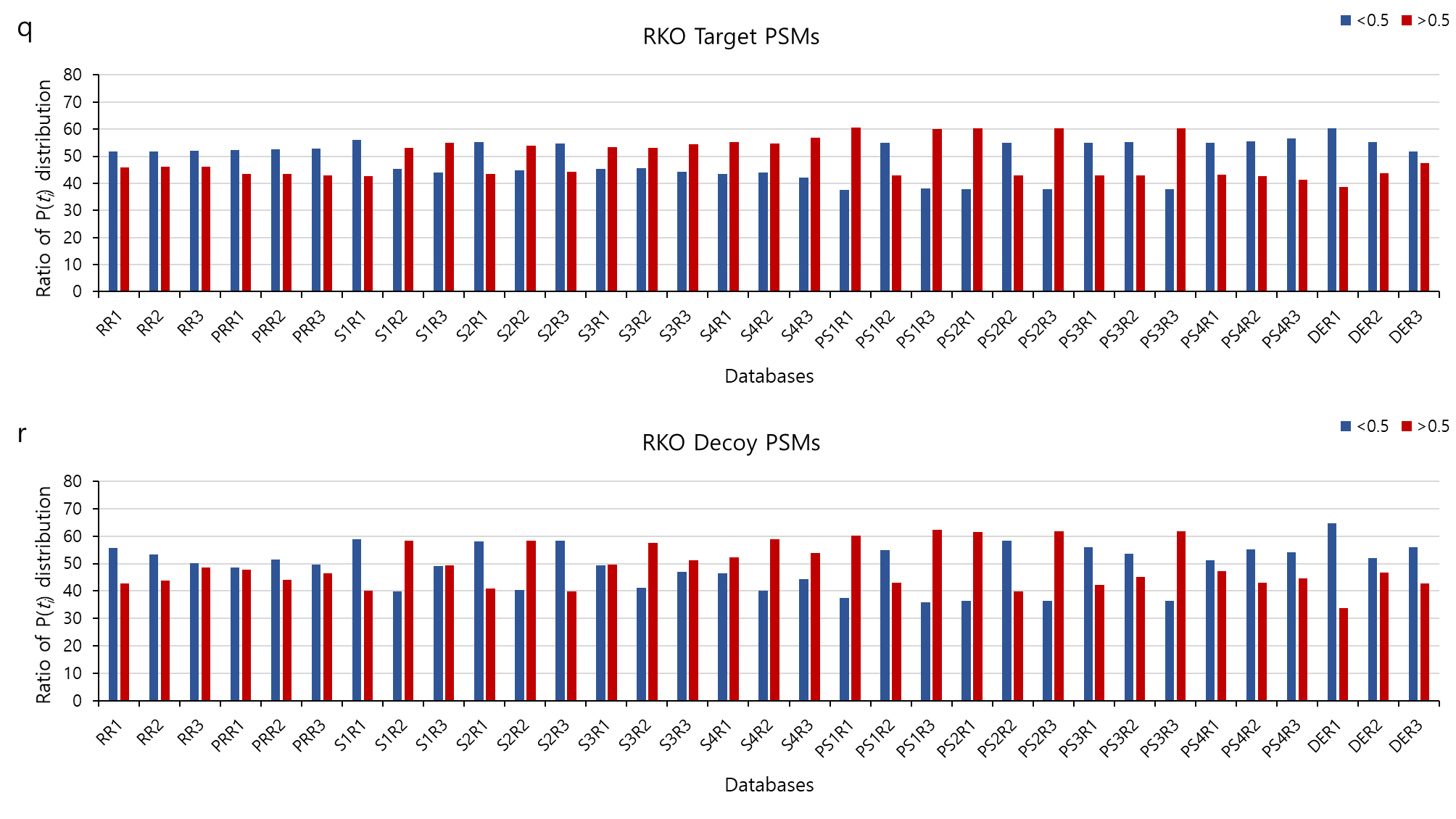
**

**
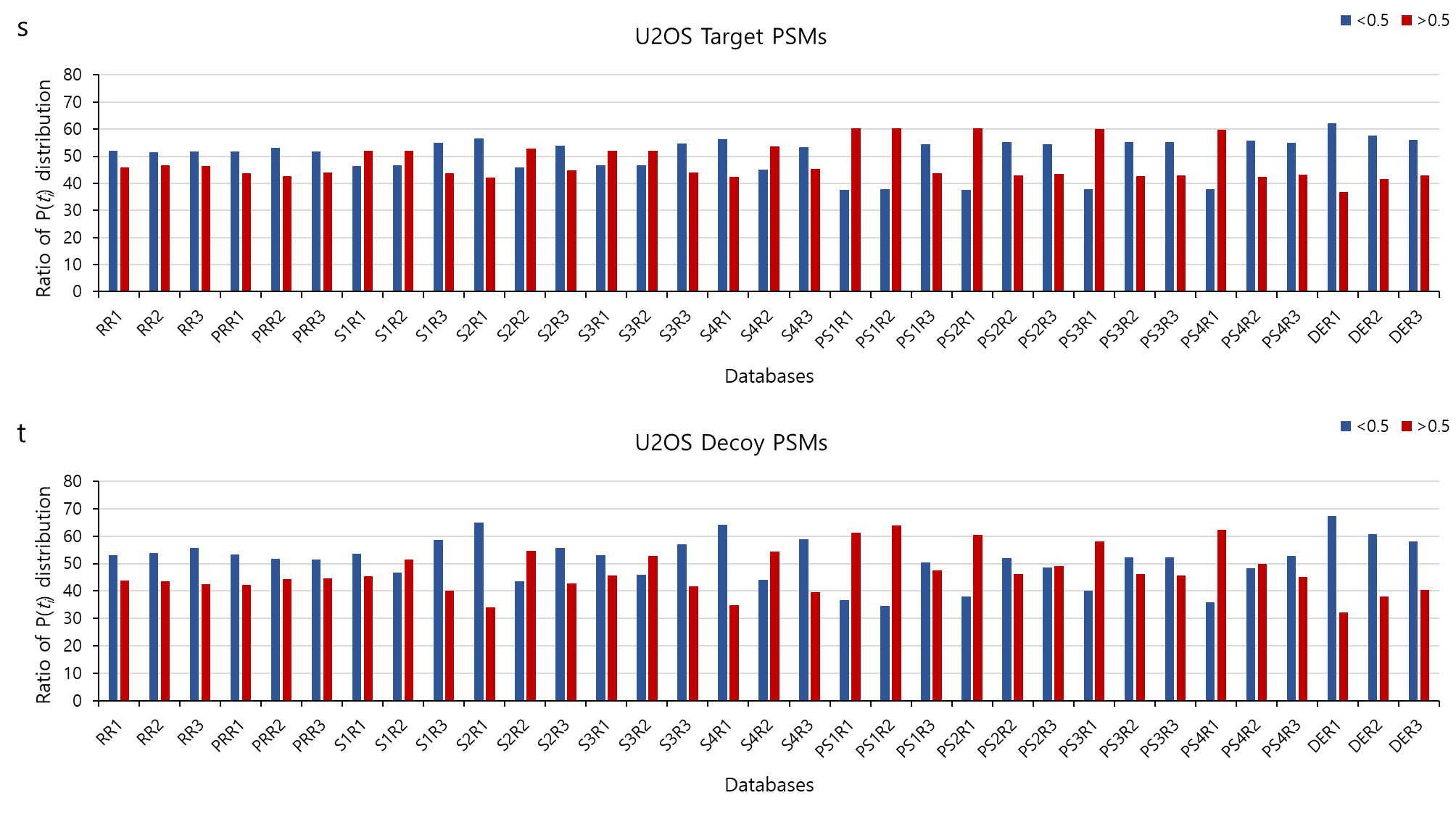
**

**Supplementary Figure 7.** Comparison of the $P(t_{i})$ distributions of the target and decoy hit rates for various databases and for the dataset here. The blue bar shows the ratio of spectra for which $P(t_{i})$ < 0.5 among all spectra at a 1% FDR threshold. The red bar shows the ratio of spectra for which $P(t_{i})$ > 0.5 among all spectra at a 1% FDR threshold. A549 (a, b), GAMG (c, d), HeLa (e, f), HepG2 (g, h), Jurkat (i, j), K562 (k, l), LnCap (m, n), MCF7 (o, p), RKO (q, r), U2OS (s, t). Each dataset is shown with the target hit rate at a 1% FDR threshold and the decoy hit rate at a 1% FDR threshold. For example, A549 (a, b): (a) A549 target hits, and (b) A549 decoy hits.
